# ND630 controls ACACA and lipid reprogramming in prostate cancer by regulating the expression of circKIF18B_003

**DOI:** 10.1186/s12967-023-04760-w

**Published:** 2023-12-04

**Authors:** Yu-Peng Wu, Wen-Cai Zheng, Qi Huang, Xu-Yun Huang, Fei Lin, Zhi-Bin Ke, Qi You, Qing-Shui Zheng, Yong Wei, Xue-Yi Xue, Ning Xu

**Affiliations:** 1 Department of Urology, Urology Research Institute, The First Affiliated Hospital, Fujian Medical University, 20 Chazhong Road, Fuzhou, 350005 China; 2 Department of Urology, National Region Medical Centre, Binhai Campus of the First Affiliated Hospital, Fujian Medical University, Fuzhou, 350212 China; 3 Fujian Key Laboratory of Precision Medicine for Cancer, The First Affiliated Hospital, Fujian Medical University, Fuzhou, 350005 China

**Keywords:** CircRNAs, Prostate cancer, ACACA, ND630

## Abstract

**Background:**

ND630 is believed to be a new therapy pharmacologic molecule in targeting the expression of ACACA and regulating the lipid metabolism. However, the function of ND630 in prostate cancer remains unknown. KIF18B, as an oncogene, plays a vital role in prostate cancer progression. circKIF18B_003 was derived from oncogene KIF18B and was markedly overexpressed in prostate cancer tissues. We speculated that oncoprotein KIF18B-derived circRNA circKIF18B_003 might have roles in prostate cancer promotion. The aim of this study was to validate whether ND630 could control ACACA and lipid reprogramming in prostate cancer by regulating the expression of circKIF18B_003.

**Methods:**

RT-qPCR was used to analyze the expression of circKIF18B_003 in prostate cancer cell lines and prostate cancer samples. circKIF18B_003 expression was modulated in prostate cancer cells using circKIF18B_003 interference or overexpression plasmid. We examined the function and effects of circKIF18B_003 in prostate cancer cells using CCK-8, colony formation, wound healing, and Transwell invasion assays and xenograft models. Fluorescence in situ hybridization (FISH) was performed to evaluate the localization of circKIF18B_003. RNA immunoprecipitation (RIP), RNA pull down, and luciferase reporter assay were performed to explore the potential mechanism of circKIF18B_003.

**Results:**

The function of ND630 was determined in this study. circKIF18B_003 was overexpressed in prostate cancer tissues, and overexpression of circKIF18B_003 was associated with poor survival outcome of prostate cancer patients. The proliferation, migration, and invasion of prostate cancer cells were enhanced after up-regulation of circKIF18B_003. circKIF18B_003 is mainly located in the cytoplasm of prostate cancer cells, and the RIP and RNA pull down assays confirmed that circKIF18B_003 could act as a sponge for miR-370-3p. Further study demonstrated that up-regulation of circKIF18B_003 increased the expression of ACACA by sponging miR-370-3p. The malignant ability of prostate cancer cells enhanced by overexpression of circKIF18B_003 was reversed by the down-regulation of ACACA. We found that overexpression of circKIF18B_003 was associated with lipid metabolism, and a combination of ND-630 and docetaxel markedly attenuated tumor growth.

**Conclusion:**

ND630 could control ACACA and lipid reprogramming in prostate cancer by regulating the expression of circKIF18B_003. ND630 and circKIF18B_003 may represent a novel target for prostate cancer.

**Supplementary Information:**

The online version contains supplementary material available at 10.1186/s12967-023-04760-w.

## Background

Prostate cancer (PCa) is the second most common malignancy in males globally [1]. It is estimated that PCa is the fifth leading cause of death among men all over the world, with approximately 375,000 men dying every year [2]. PCa is a very heterogeneous disease, the clinical presentation of PCa in males varies greatly [3]. Some Prostate patients are localized and indolent diseases, the other are lethal metastatic diseases with rapid progression [4–6]. Nowadays, because of relapse or late diagnosis of PCa, the prognosis of patients remains dismal. At present, the widely used diagnostic assays, like prostate-specific antigen, lack adequate specificity and sensitivity to diagnose or predict the prognosis of PCa. Therefore, current commonly used biomarkers for monitoring tumor growth still have great limitations.

Circular RNAs (circRNAs) are a kind of endogenous non-coding RNA that have a covalently closed-loop structure. This structure differentiates circular RNAs from linear RNA molecules, which have a 5′ cap and a 3′ poly (A) tail [7, 8]. The characteristics of circRNAs, including high stability, evolutionary conservation, and abundance, make them suitable for use as novel biomarkers for cancer in clinical practice [9]. For instance, He et al. [10] demonstrated that circRNA_100395 exhibits tumor suppressive effectives and a potential therapeutic target for PCa. Xu et al. [11] revealed that androgen receptor regulates PCa cell invasion via inhibiting circRNAs-51217 expression through regulating ADAR2 expression.

Cancer cells undergo metabolic reprogramming to accommodate the biosynthetic needs for supporting continuous cell growth and proliferation [12]. Elevated rates of de novo fatty acid synthesis, which is a metabolic feature of tumor cells, play an essential role in regulating the progression of tumor cells. Acetyl-CoA carboxylase alpha (ACACA) is the rate-limiting enzyme of fatty acid synthase (FAS), which functions in catalyzing the carboxylation of CO_2_ and conversion of acetyl-CoA into malonyl-CoA [13]. Therefore, ACACA plays a key role in regulating fatty acid formation. ND-630, a compound known to inhibit ACACA or Acetyl-CoA carboxylase alpha, an enzyme that plays a crucial role in fatty acid synthesis and has been associated with various cancers. ND-630 has shown promise in attenuating the growth of cancer cells by disrupting lipid metabolism [14]. Considering that circKIF18B_003 might influence the same metabolic pathway, it's plausible that the combined action of ND-630 and circKIF18B_003 could offer a novel therapeutic approach for PCa.

Kinesin family member 18B (KIF18B), an ATPase with essential involvement in cell division, has been implicated in chromosomal separation and pairing during mitosis [15, 16]. We previously reported the role of KIF18B in the proliferation and invasion of PCa in vitro and in vivo [16]. Therefore, we speculated that KIF18B derived circRNA, circKIF18B_003, may play an important part in the development of PCa. We discovered a new circRNA derived from KIF18B termed circKIF18B_003 and found that this circRNA was markedly upregulated in PCa tissues compared with levels in matched normal tissues. High circKIF18B_003 expression level was positively associated with worse outcome of PCa. Mechanistically, elevated circKIF18B_003 upregulated ACACA expression to promote PCa cell malignant activity and lipid metabolism reprogramming by sponging microRNA-370-3p (miR-370-3p). The results of this study demonstrated that circKIF18B_003 increase the progression of PCa and suggests that it might be a viable therapeutic target. The objective of this research was to figure out whether ND630 could control ACACA and lipid reprogramming in prostate cancer by regulating the expression of circKIF18B_003.

## Methods

### Tissue sample collection

Our study utilized a total of 78 prostate cancer (PCa) tissue samples and 23 normal prostatic tissue samples. The 23 normal tissue samples were obtained from regions adjacent to the tumor in the same patients, ensuring that they were not affected by the disease. All tissue samples were collected following the surgical removal of the tumors. These were systematically collected from patients at the Department of Urology of the First Affiliated Hospital of Fujian Medical University. The patients included in this study had to meet the following criteria: (1) they had not undergone any form of chemotherapy or radiotherapy prior to the tissue collection, (2) they had undergone radical prostatectomy, and the diagnosis of PCa was independently confirmed by two pathologists, (3) they had provided their informed consent for their tissue samples to be used in this study.

Upon collection, tissue samples were immediately flash-frozen in liquid nitrogen to maintain their integrity and stored at − 80 °C until further analysis. Histopathological confirmation was achieved by fixing the tissues in 10% buffered formalin, embedding in paraffin, and staining with Hematoxylin and Eosin. The presence of PCa or normal cells was ascertained by two independent pathologists. For molecular analyses, such as gene expression profiling, tissues were homogenized and RNA was extracted using a standardized protocol. The entire process was carried out under stringent sterile conditions to prevent any possible contamination.

### The Cox's proportional hazard regression model

The Cox's proportional hazard regression model was utilized in our study to identify independent prognostic factors affecting the time to biochemical recurrence in prostate cancer patients. This model is particularly useful in estimating the impact of multiple covariates on survival time, while also accounting for potential confounding effects. In our study, the Cox model was fitted with the following covariates: Age, Prostate Volume, Prostate Specific Antigen (PSA) levels, Gleason Score, Pathological Tumor Stage (pT stage), and the expression level of circKIF18B_003. Age and Prostate Volume were included as general patient characteristics that could potentially influence the disease course. PSA levels, Gleason Score, and pT stage are established clinical parameters in prostate cancer prognosis. Using the Cox model, we aimed to determine the independent effect of each of these factors on the time to biochemical recurrence, thereby gaining a more comprehensive understanding of their roles in prostate cancer progression.

### Cell culture, constructs, transfection, and establishment of stable cell lines

Cell lines including PC-3, DU145, LNCaP, RWPE-1, and HEK-293T were obtained from Procell Life Science & Technology Co., Ltd (Wuhan, China). The culture conditions varied for each cell line. PC-3 cells were maintained in F-12K medium (Gibco BRL, Grand Island, NY, USA) supplemented with 10% fetal bovine serum (FBS, Gibco, Australia) at a density of 5 × 10^5 cells per well in 6-well plates. LNCaP cells were cultured in RPMI-1640 medium (Gibco BRL) enriched with 10% FBS at a seeding density of 4 × 10^5 cells per well. DU145 and HEK-293 T cells were cultured in Minimum Essential Medium (MEM) and Dulbecco's Modified Eagle Medium (DMEM) respectively, both supplemented with 10% FBS, with a cell seeding density of 5 × 10^5 cells per well. RWPE-1 cells were cultured in Keratinocyte Serum Free Medium (KSFM, Gibco). All cells were incubated in a humidified atmosphere at 37 °C with 5% CO2.

For RNA interference and overexpression experiments, small hairpin RNAs (shRNAs) and complementary DNA (cDNA) plasmids of circKIF18B_003, small interfering RNA (siRNA) targeting ACACA, and miR-370-3p mimics were procured from Shanghai GenePharma Co., Ltd (Shanghai, China). The sequences used were as follows:

shRNA1 for circKIF18B_003: 5′-GAGATCAGGCTCTCGGTGTCA-3′

shRNA2 for circKIF18B_003: 5′-GATCAGGCTCTCGGTGTCACC-3′

shNC: 5′-TTCTCCGAACGTGTCACGT-3′

siACACA-1: 5′-GCUUCUACUUUCUGGAAUUTT-3′

siACACA-2: 5′-GCUCAUACACUUCUGAAUATT-3′

siACACA-3: 5′-GCAGCUAUGUUCAGAGAAUTT-3′.

Negative control siRNA: 5′-UUCUCCGAACGUGUCACGUTT ACGUGACACGUUCGGAGAATT-3′.

miR-370-3p mimic: 5′-GCCUGCUGGGGUGGAACCUGGUCAGGUUCCACCCCAGCAGGCUU-3′.

Transfections were performed using Lipofectamine 2000 (Invitrogen, Carlsbad, CA), following the manufacturer's protocol. Transfections were performed using Lipofectamine 2000 (Invitrogen, Carlsbad, CA), following the manufacturer's protocol. Specifically, cells at 70% confluence were transfected with 50 nM of each construct. Following 48 h of transfection, cells were harvested for subsequent experiments. Stable cell lines expressing specific constructs were established by selecting cells with appropriate antibiotics for 2 weeks post-transfection. Resistant colonies were expanded and construct expression was validated by RT-PCR or Western blotting.

### Quantitative real-time polymerase chain reaction (qRT-PCR) and western blot assays

We conducted qRT-PCR and Western blot assays in accordance with the methodologies previously detailed in our earlier study [16]. For qRT-PCR, total RNA was isolated from cells using TRIzol reagent (Invitrogen, Carlsbad, CA) as per manufacturer's instructions. RNA concentration was measured using a NanoDrop Spectrophotometer (Thermo Fisher Scientific, Waltham, MA). Subsequently, 1 μg of total RNA was reverse-transcribed into cDNA using the High Capacity cDNA Reverse Transcription Kit (Applied Biosystems, Foster City, CA). qRT-PCR reactions were performed using the SYBR Green PCR Master Mix (Applied Biosystems). The specific primer sequences used for qRT-PCR are listed in Additional file 4: Table S1. The relative expression of each gene was calculated using the 2^-ΔΔCt method, with GAPDH serving as the internal control. For Western blot assays, cells were lysed in RIPA buffer supplemented with protease and phosphatase inhibitor cocktails (Thermo Fisher Scientific). Protein concentrations were determined using the BCA Protein Assay Kit (Thermo Fisher Scientific). Equal amounts of protein were separated by SDS-PAGE and then transferred onto PVDF membranes (Millipore, Billerica, MA). The membranes were blocked with 5% non-fat milk and probed with primary antibodies overnight at 4 °C. The specific antibodies used are listed in Additional file 5: Table S2. The membranes were then incubated with HRP-conjugated secondary antibodies, and the bands were visualized using the ECL detection system (GE Healthcare, Chicago, IL). The intensity of the bands was quantified using ImageJ software (NIH, Bethesda, MD), with β-actin or GAPDH serving as the loading control.

### Immunohistochemistry (IHC) and fluorescence in situ hybridization (FISH) assays

For immunohistochemistry studies, we closely adhered to the protocol delineated in our previous research [16]. Briefly, formalin-fixed paraffin-embedded tissue sections (4 µm thick) were deparaffinized and rehydrated. Antigen retrieval was performed in citrate buffer (pH 6.0) using a microwave. Endogenous peroxidase activity was blocked with 3% hydrogen peroxide. Sections were then incubated with primary antibodies (listed in Additional file 5: Table S2) overnight at 4 °C. The slides were washed and incubated with biotinylated secondary antibodies, followed by incubation with streptavidin–horseradish peroxidase. Diaminobenzidine was used as the chromogen, and the sections were counterstained with hematoxylin. The immunostaining intensity was evaluated by a pathologist blinded to the study.

Fluorescence in situ hybridization (FISH) assay was performed to visualize the localization of CircKIF18B 003 and miR-370-3p. The specific Cy3-labeled probe for CircKIF18B 003 and FAM-labeled probe for miR-370-3p were procured from Shanghai GenePharma Co., Ltd (Shanghai, China). The sequences of the probes were as follows:

CircKIF18B 003 probe: 5′- GACACCGAGAGCCTGATCTCCTTGGCTGTGGTGACACCGAGAGCCTGATCTGCCATCACTGTGGTGACACCGAGA-3′.

miR-370-3p probe: 5′-ACCAGGTTCCACCCCAGCAGGC-3′.

Negative Control (NC) probe: 5′-TGCTTTGCACGGTAACGCCTGTTTT-3′.

After prehybridization, the probes were hybridized with the target sequences in the presence of pre-prepared hybridization buffer. Post-hybridization, the slides were washed to remove unbound probes. Nuclei were counterstained with 4′6-Diamidino-2-phenylindole (DAPI) (Solarbio, Beijing, China). The cells were examined under a confocal microscope, and the fluorescence intensity was analyzed using ImageJ software (NIH, Bethesda, MD). The FISH signals were quantified by counting the number of positive cells in five random fields per slide.

### Cell Counting Kit-8 (CCK-8), colony formation, wound healing migration, and Transwell invasion assays

We performed Cell Counting Kit-8 (CCK-8), colony formation, wound healing migration, and Transwell invasion assays as previously described in our earlier study [16]. For the CCK-8 assay, cells were seeded in 96-well plates at a density of 5 × 10^3 cells per well and cultured for 24, 48, 72, and 96 h. 10 µL of CCK-8 solution (Dojindo Molecular Technologies, Kumamoto, Japan) was added to each well and incubated for 2 h at 37 °C. The optical density (OD) at 450 nm was measured using a microplate reader to estimate the number of viable cells. In the colony formation assay, cells were seeded in 6-well plates at approximately 500 cells per well and incubated at 37 °C for 2 weeks. The colonies were fixed with methanol and stained with 0.1% crystal violet. The number of colonies, defined as > 50 cells/colony, was manually counted. The wound healing migration assay was initiated by seeding cells into 6-well plates and allowing them to grow until 90% confluency. A sterile 200 µL pipette tip was used to create a straight scratch in the cell monolayer. The cells were washed to remove cellular debris and incubated in serum-free medium. The wound closure was observed and imaged at 0 and 24 h using an inverted microscope. For Transwell invasion assays, 5 × 10^4 cells in serum-free medium were placed in the upper chamber of a Transwell insert (Corning, Corning, NY) pre-coated with Matrigel (BD Biosciences, Franklin Lakes, NJ). The lower chamber was filled with medium supplemented with 10% fetal bovine serum as a chemoattractant. After 24 h of incubation at 37 °C, the cells on the upper surface of the membrane were removed, and the cells on the lower surface were fixed with methanol and stained with 0.1% crystal violet. The invaded cells were imaged and counted in five random fields per well under a microscope.

### Circular RNA immunoprecipitation (circRNA RIP) assay

The circRNA RIP assay was carried out using a biotin-labeled circKIF18B_003 probe generously supplied by Shanghai GenePharma Co., Ltd (Shanghai, China). The sequence for the circKIF18B_003 RNA pull-down probe was as follows: 5′-CACUGUGGUGACACCGAGAGCCUGA-3′. Initially, PC-3 cells were seeded in a 10 cm dish and cultured for 48 h. The biotin-labeled circKIF18B_003 probe and a nonspecific control probe (both at 200 nM) were then transfected into PC-3 cells using Lipofectamine 2000 (Invitrogen, Carlsbad, CA) according to the manufacturer's instructions. The cells were incubated at 37 °C in a 5% CO2 environment for 24 h post-transfection. Subsequently, the circKIF18B_003-overexpressing PC-3 cells were washed with Phosphate-buffered saline (PBS) and then fixed with 1% formaldehyde for 10 min at room temperature to cross-link proteins and RNA. Glycine was added to quench the fixation reaction. The cells were then lysed in co-immunoprecipitation (CO-IP) buffer containing protease and RNase inhibitors, sonicated to shear the DNA, and centrifuged at 12,000*g* for 10 min at 4 °C. The supernatant was collected and a 50 µL aliquot was set aside as input. The remaining supernatant was then incubated with probe-streptavidin-dynabeads (Thermo Fisher Scientific, Waltham, MA) complex at 30 °C for 12 h to capture the circRNA-protein complexes. The beads were then washed to remove non-specifically bound materials. The bound circRNA-protein complexes were eluted from the beads by treatment with proteinase K and lysis buffer at 55 °C for 30 min. The eluted RNA was extracted using phenol–chloroform-isoamyl alcohol and precipitated with ethanol. The recovered RNA was then subjected to qRT-PCR to analyze the associated proteins. The data were normalized to the input.

### RNA pull-down assay

For the RNA pull-down assay, we employed the PureBinding™ RNA–Protein Pull-Down Kit (Geneseed Biotech Co., Ltd., Guangzhou, China), adhering to the protocols described in previous studies [17, 18]. We utilized a biotin-labeled miR-370-3p probe, generously provided by Shanghai GenePharma Co., Ltd (Shanghai, China). The sequence for the miR-370-3p RNA pull-down probe was as follows: 5′-CAGGUCACGUCUCUGCAGUUAC-3′. The circKIF18B_003-overexpressing cells were initially washed with Phosphate-buffered saline (PBS) and subsequently fixed with 1% formaldehyde for 10 min at room temperature to cross-link RNA and protein. The fixation was quenched by adding glycine. The cells were then lysed in Co-IP buffer containing protease and RNase inhibitors, followed by sonication to shear the DNA, and centrifugation at 12,000*g* for 10 min at 4 °C to pellet the cell debris. The supernatant was carefully collected, and a 50 µL aliquot was set aside to serve as the input sample. The remaining supernatant was incubated with the pre-washed streptavidin-dynabeads (Thermo Fisher Scientific, Waltham, MA) bound to the biotin-labeled miR-370-3p probe at 30 °C for 12 h. This allowed the specific binding of miR-370-3p and its associated proteins to the beads. The beads were then washed to remove unbound materials. The bound miR-370-3p-protein complexes were eluted from the beads by incubation with proteinase K and lysis buffer at 55 °C for 30 min. The eluted RNA was extracted using phenol–chloroform-isoamyl alcohol, precipitated with ethanol, and subjected to qRT-PCR for analysis of the associated proteins. The data were normalized to the input sample.

### RNA immunoprecipitation (RIP) assay

The RIP assay was conducted using an RNA Immunoprecipitation kit (Geneseed Biotech Co., Ltd., Guangzhou, China), following the methodology detailed in previous studies [19, 20]. For the assay, circKIF18B_003-overexpressing cells were first washed with Phosphate-buffered saline (PBS) to remove any residual medium. The cells were then lysed in RIPA buffer (containing protease and RNase inhibitors) to release the intracellular content. This was followed by centrifugation at 12,000*g* for 10 min at 4 °C to pellet the cell debris. The supernatant, containing the cell lysate, was transferred to a new tube and kept on ice. The cell lysate was then incubated overnight at 4 °C on a rotating device with 5 µg of anti-AGO2 antibody or negative control IgG. The next day, the lysate was incubated with Protein A + G agarose beads for 2 h at 4 °C to capture the antibody-bound RNA–protein complexes. After the incubation, the beads were precipitated by centrifugation and washed thoroughly to remove unbound material. The RNA–protein complexes bound to the beads were then eluted by incubation with proteinase K at 55 °C for 30 min. The RNA was then extracted from the eluate using phenol–chloroform-isoamyl alcohol and precipitated with ethanol. The isolated RNA was reverse transcribed into cDNA using SuperScript™ IV Reverse Transcriptase (Invitrogen, Carlsbad, CA) following the manufacturer's instructions. The resulting cDNA was used as a template for quantitative real-time PCR (qRT-PCR) to determine the enrichment of circKIF18B_003 in the immunoprecipitates. The qRT-PCR was performed using SYBR Green (Bio-Rad, Hercules, CA) on a real-time PCR system. The data were normalized to the input sample.

### Firefly luciferase (luciferase) reporter assay

The Firefly Luciferase Reporter Assay was carried out with the assistance of reporter vectors that were kindly synthesized and provided by Shanghai GenePharma Co., Ltd (Shanghai, China). The vectors were designed to carry either the wild-type or mutant sequences of circKIF18B_003-miR-370-3p and ACACA-miR-370-3p binding sites.

The sequences for the wild-type and mutant vectors were as follows:

circKIF18B_003-miR-370-3p Wild-Type Vector: CCCATGCCATCTTCCAGATCTTTGTGAAGCAGCAGGACCGGGTTCCAGGACTGACCCAGG

circKIF18B_003-miR-370-3p Mutant Vector: CCCATGCCATCTTCCAGATCTTTGTGAAGGTCGTCCACCGGGTTCCAGGACTGACCCAGG

ACACA-miR-370-3p Wild-Type Vector: GTTCTACTCTCTTCCCCAGAGTGTAGACAGGCAGCAGGTCTCCCCACAGCTCTGAAAGGA

ACACA-miR-370-3p Mutant Vector: GTTCTACTCTCTTCCCCAGAGTGTAGACAGGGTCGTCCTCTCCCCACAGCTCTGAAAGGA.

The luciferase assays were executed in accordance with the manufacturer's instructions using the Dual-Lumi™ Luciferase Assay Kit (Beyotime Biotechnology, Shanghai, China). Cells were seeded in 96-well plates at a density of 1 × 10^4 cells per well and allowed to adhere overnight. The following day, the cells were co-transfected with either the miR-370-3p mimics or the negative control and the appropriate luciferase reporter vector using Lipofectamine 2000 reagent (Invitrogen) as per the manufacturer's protocol. After 48 h of transfection, the luciferase activity was measured using a luminometer. The firefly luciferase activity was normalized to the Renilla luciferase activity to account for differences in transfection efficiency. The experiment was performed in triplicate and the data were presented as the mean ± standard deviation (SD).

### In vivo assays

Six-week-old male nude mice were procured from Shanghai SLAC Laboratory Animal Co., Ltd (Shanghai, China). The circKIF18B_003-overexpressing PC-3 cells (6 × 10^6 cells/mouse suspended in 100 μL of serum-free medium) were subcutaneously implanted into the right flank of each mouse. We divided the mice into four groups, each containing three mice: (1) the PBS control group, (2) the ND-630 treatment group, (3) the combined ND-630 and siACACA1 treatment group, and (4) the combined ND-630 and siACACA2 treatment group. The treatment regimens were modeled after a previous study [21]. One week post-implantation of the circKIF18B_003-overexpressed PC-3 cells, the treatment was initiated. ND-630 was first dissolved in dimethyl sulfoxide (DMSO) to create a stock solution, which was then further diluted 1:50 in PBS to achieve the desired working concentration. In the ND-630 treatment groups, mice were administered ND-630 daily via oral gavage for a total of 4 weeks. After the treatment period, the mice were humanely euthanized, and the tumors were carefully excised. Both the weight and volume of the tumors were recorded for each mouse. The tumor volume was calculated using the formula π/6 × ab^2, where 'a' represents the length and 'b' is the width of the tumor (with 'a' greater than 'b'). All procedures were conducted following ethical guidelines and were approved by the institution's animal care and use committee. Measurements and observations were made by personnel blinded to the treatment groups to minimize bias. Data were analyzed using statistical methods, and results were reported as the mean ± standard deviation.

### Cell lipid profile analysis

The cellular lipid profile, specifically the levels of triglyceride (TG) and fatty acid (FA), were evaluated using the Human TG and FA Enzyme-Linked Immunosorbent Assay (ELISA) Kits (Meibiao Biotechnology, Jiangsu, China). The assays were conducted in strict adherence to the manufacturer's instructions. The cells were first cultured until reaching 70–80% confluence in appropriate culture medium. Following this, they were harvested, washed twice with cold PBS, and then lysed using a suitable lysis buffer provided in the kit. The cell lysates were then centrifuged at 12,000*g* for 10 min at 4 °C. The supernatant was collected carefully to avoid disturbing the pellet. The supernatant, containing the cellular lipids, was then used for the ELISA assays. For each assay, standards and samples were added to their respective wells of the ELISA plate, which were pre-coated with antibodies specific to human TG or FA. After an incubation period to allow for antigen–antibody binding, the plate was washed to remove any unbound substances. Next, an enzyme-linked secondary antibody was added to each well and incubated. The plate was washed again to remove any unbound secondary antibodies. The substrate solution was then added to induce a color reaction, the intensity of which is proportional to the concentration of TG or FA in the sample. After the addition of stop solution, the absorbance of each well was measured using a microplate reader at a wavelength of 450 nm. The concentration of TG and FA in the samples was then calculated by comparing the absorbance of the samples to a standard curve. All measurements were made in triplicate to ensure accuracy and consistency. The results were then analyzed statistically and presented as mean ± standard deviation.

### Seahorse XF24 assays

Seahorse XF24 assays were conducted to assess the metabolic characteristics of the cells, specifically their oxygen consumption rate (OCR) and extracellular acidification rate (ECAR), indicative of oxidative phosphorylation and glycolysis respectively. These assessments were performed using the Seahorse XF24 Extracellular Flux Analyzer (Agilent Technologies, Santa Clara, CA, USA). The cells were first cultured under appropriate conditions until they reached 70–80% confluence. They were then seeded in a Seahorse XF24 cell culture microplate at a density of approximately 2 × 10^4 cells per well and allowed to adhere overnight, ensuring optimal cell health and attachment. On the day of the assay, the culture medium was replaced with Seahorse XF base medium (Agilent Technologies), supplemented according to the manufacturer's instructions and adjusted to pH 7.4. The cells were then incubated in a non-CO2 atmosphere for one hour to allow temperature and pH equilibration before the assay. The Seahorse XF24 Analyzer was programmed to perform multiple measurement cycles, each consisting of mixing, waiting, and measuring periods to ensure accurate readings. The OCR and ECAR were measured under basal conditions and in response to the sequential addition of specific metabolic inhibitors, such as oligomycin, FCCP (carbonyl cyanide-p-trifluoromethoxyphenylhydrazone), and a mixture of rotenone and antimycin A. These compounds are typically used to evaluate mitochondrial respiration and glycolytic capacity. All experiments were performed in triplicate to ensure the accuracy and reproducibility of the results. The data was normalized to cell number, determined by a parallel cell count, and reported as the mean ± standard deviation. Statistical comparisons between groups were performed using appropriate statistical tests, and a P-value of less than 0.05 was considered statistically significant.

### Determination of glucose incorporation into de novo fatty acid synthesis by liquid chromatography‐mass spectrometry analysis

A population of 5 × 10^6 PC-3 or DU145 cells was subjected to two wash cycles with PBS, followed by a 24-h incubation in a 10 mL culture medium. This medium was composed of 10% FBS, 90% glucose-free DMEM, and 25 mm d-glucose-13C6 (MCE, HY-B0389A). Post-incubation, cells were washed twice with precooled PBS, and then combined with 1 mL of precooled methanol. The cell mixture was then collected through scraping in preparation for the subsequent Liquid Chromatography-Mass Spectrometry (LC–MS) analysis. The LC–MS analysis was performed using an ACQUITY UPLC HSS T3 column (Waters, Ireland) for reversed-phase chromatographic procedures. An Agilent 1290 II ultra-performance liquid chromatography system (Agilent Technologies) in tandem with a 5600 Triple TOF Plus quadrupole-TOF MS instrument (AB SCIEX) was utilized for metabolome data acquisition. The information-dependent acquisition mode was selected for MS/MS metabolite analysis. Data collection and processing were conducted using Analyst™ TF 1.7.1 software (AB SCIEX, Canada).

### Bioinformatics analysis

Firstly, circBase [22] database (http://circrna.org/) was used in this study to inquire about the specific data of circKIF18B_003, including the RNA sequences, and the relationships between the circKIF18B_003 and its host gene KIF18B, the construct and the length of circKIF18B_003. StarBase [23] of The Encyclopedia of RNA Interactomes (ENCORI) is a database that mainly focuses on microRNA (miRNA)-target interactions (https://starbase.sysu.edu.cn/). CircBank [24] is a comprehensive database of human circRNA, which could be used to predict the binding miRNA of circRNA (http://www.circbank.cn/). NIH_Interactome database [25], also called CircInteractome database, could be used to identify miRNAs targeting a circRNA (https://circinteractome.nia.nih.gov/). These three databases were used to predict the potential targeted miRNAs of circKIF18B_003. Kyoto Encyclopedia of Genes and Genomes [26] (KEGG, https://www.kegg.jp/kegg/) was used to predict the potential pathways of the identified mRNAs.

### Statistical analysis

Statistical analyses were meticulously executed using the Statistical Package for the Social Sciences (SPSS) software, version 19.0 (SPSS, Inc., Chicago, IL, USA). The data are represented as mean ± standard deviation (SD). To compare groups, multiple statistical tests were employed. The Student's t-test was used for comparing means between two groups, while the Chi-squared test was utilized for categorical variables. The Mann–Whitney U test was applied for comparing non-normally distributed continuous data between two groups, whereas the Kruskal–Wallis test served to compare such data across more than two groups. For comparisons of means across more than two groups, one-way analysis of variance (ANOVA) was performed.

Survival outcomes were analyzed using Kaplan–Meier survival curves, with differences between curves assessed by the log-rank test. To determine independent prognostic factors, Cox's proportional hazard regression model was applied. For the Cox regression analyses, variables with a P-value of less than 0.05 in the univariate analysis were included in the multivariate analysis. Hazard ratios (HRs) and 95% confidence intervals (CIs) were calculated to quantify the impact of each variable on biochemical recurrence survival. The Receiver Operating Characteristic (ROC) curve analysis was used to determine the predictive value of circKIF18B_003 expression for PCa. The area under the curve (AUC) was calculated, with an AUC of 1 indicating perfect predictive power, and an AUC of 0.5 indicating a prediction no better than chance.

All tests were two-tailed, and a P-value of less than 0.05 was deemed to denote statistical significance. The consistency and reliability of results were ensured by conducting each test with an appropriate sample size, derived from power analysis. In addition, the assumptions for each statistical test were verified before their application. Multiple testing correction was applied where necessary to reduce the probability of Type I errors.

### CircKIF18B_003 circRNA identification

**Fig. 1:**
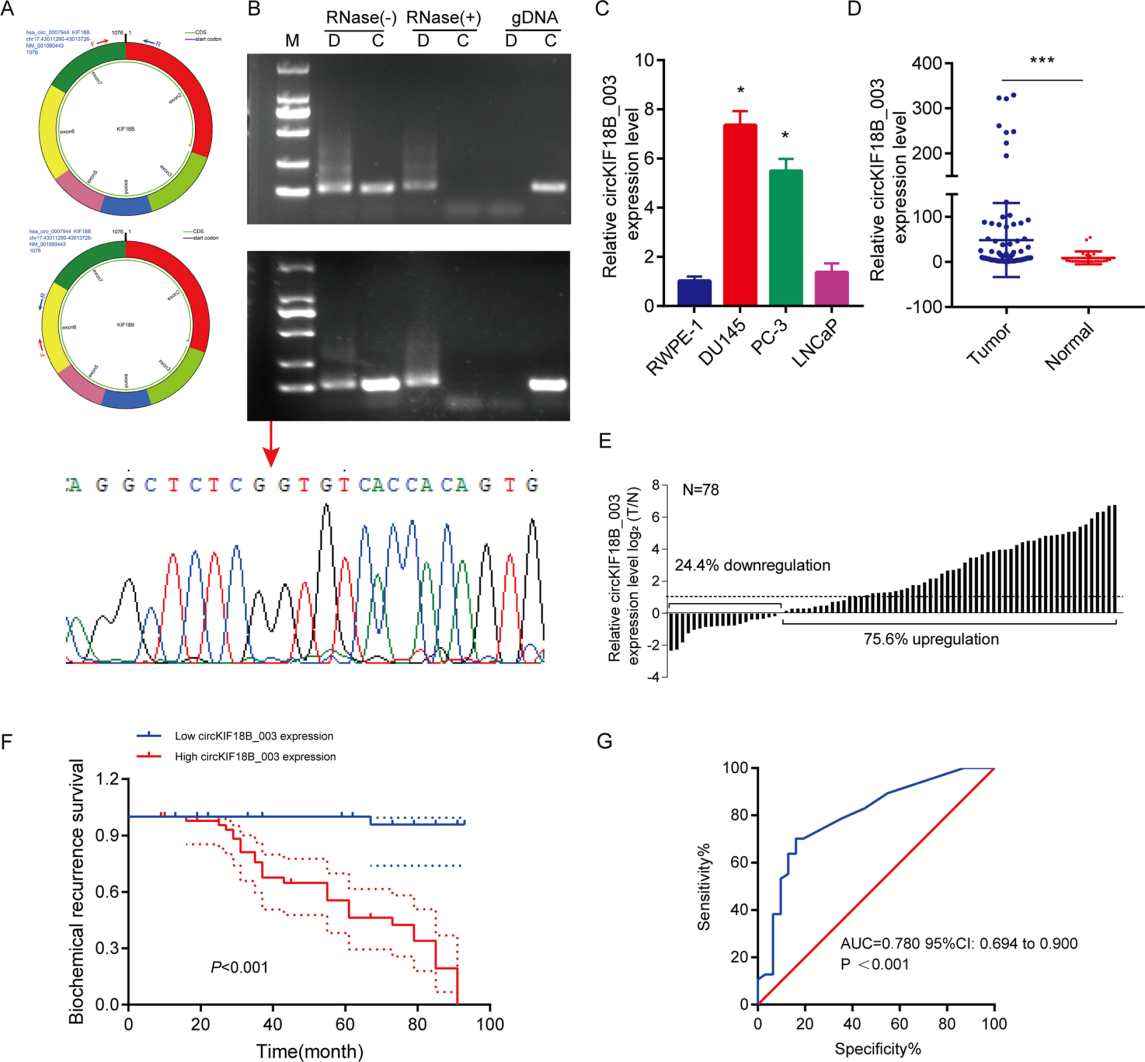
Analyses of circKIF18B_003 expression in PCa. **A** Schematic illustration of circKIF18B_003 formation via circularization of exons 2–7 of the KIF18B gene. Divergent (upper panel) and convergent (lower panel) primers were designed to verify circKIF18B_003. **B** Using RT-qPCR and agarose gel electrophoresis, the existence of circKIF18B 003 was confirmed. Total RNA extracted from DU145 cells (upper panel) or PC-3 cells (middle panel) with or wither RNase-R treatment were subjected to polymerase chain reaction. The products conducted by divergent primers of circKIF18B_003 was subsequently validated by Sanger sequencing (lower panel), and the red arrow confirmed the “head-to-tail” splicing of circKIF18B_003 in PCa cells. **C** The relative expression level of circKIF18B_003 was evaluated in the RWPE-1 cell line and different PCa cell lines, including DU145, PC-3, and LNCaP lines. **D** The relative expression of circKIF18B_003 level in 78 PCa tissues and 23 benign prostatic hyperplasia tissues was analyzed by RT-qPCR. Mann–Whitney U test was used for the statistical analyses. **E** The relative expression level of circKIF18B_003 in PCa tissue and the average expression level of circKIF18B_003 in 23 cases of benign prostatic hyperplasia tissues were compared and analyzed. **F** Kaplan–Meier method was used to demonstrate the biochemical recurrence survival curve and log-rank test was used to analyze the survival data. **G** The area under the curve in determining the efficacy of the model in predicting the biochemical recurrence survival of PCa patients. Student’s t-test was used for the statistical analyses. ^*^P < 0.05; ^***^P < 0.001

A comparison of the RNA sequences of circKIF18B_003 (located at chr17:43011290–43013726) and KIF18B from circBase revealed that circKIF18B_003 is a looped structure, consisting of exons 2–7 of the KIF18B gene, and is 1076 bp in length. To confirm these exons indeed formed circKIF18B_003, divergent and convergent primers were designed (Fig. 1A). Amplification products that were resistant to RNase-R digestion were obtained through RT-qPCR with the divergent primers (Fig. 1B). However, qPCR performed on genomic DNA with these primers yielded no products, ruling out PCR artifact or genomic rearrangement as causes for amplification. Conversely, convergent primers' PCR products from linear KIF18B mRNA were susceptible to RNase-R digestion. Sanger sequencing confirmed the circKIF18B_003 junction.

### CircKIF18B_003 analysis in prostate cancer (PCa)

Our previous work demonstrated that KIF18B is overexpressed in PCa. We thus next examined the expression of circKIF18B_003 in PCa cell lines and paired PCa tissues and corresponding adjacent normal prostatic tissues. RT-qPCR demonstrated that circKIF18B_003 levels were markedly higher in PC-3, DU145, and LNCaP cell lines than in normal prostatic epithelial RWPE-1 cells line. (Fig. 1C). circKIF18B_003 was also highly expressed in PCa tissues (Fig. 1D). The expression level of circKIF18B_003 was higher in PCa tissues and markedly higher (log2FC ≥ 1) in PCa tissues than that in corresponding normal prostatic tissues (Fig. 1E).

The relationship between circKIF18B_003 expression and clinicopathological features in PCa patients was further investigated. Prostate volume, PSA, Gleason score, and pathological T stage were significantly linked to circKIF18_003 expression (Table 1). We did observe, however, that for categories such as PSA > 10, Gleason score > 7, and pT stage T3-T4, there was a small number of patients exhibiting low circKIF18B_003 expression. Patient stratification into low and high expression groups was based on sorted average expression values as outlined in a previous study [27]. High circKIF18B_003 expression corresponded with shorter biochemical recurrence (Fig. 1F). The predictive value of circKIF18B_003 in forecasting biochemical recurrence survival in PCa patients was evaluated using ROC analysis (Fig. 1G). The resulting area under the curve was 0.780, with a 95% confidence interval ranging from 0.694 to 0.900 (P < 0.001), demonstrating a strong predictive potential. Univariate and multivariate Cox regression analyses further confirmed the prognostic accuracy of circKIF18B_003 in predicting the biochemical recurrence of PCa. Findings indicated that the Gleason score, pathological T stage, and circKIF18B_003 expression are independent risk factors for PCa patients (Table 2).

**Table 1:** Correlations between circKIF18B_003 expression and clinicopathological characteristics of prostate cancer patients

Variable	Number of Patients		p-value
	circKIF18B_003		
	Low expression	High expression	
Age, year			
≤ 70	16	17	0.177
> 70	15	30	
Prostate volume, cm^3^			
≤ 35	22	18	0.005
> 35	9	29	
PSA, ng/mL			
≤ 10	29	24	< 0.001
> 10	2	23	
Gleason score			
≤ 7	31	27	< 0.001
> 7	0	20	
pT stage			
T1–T2	30	24	< 0.001
T3–T4	1	23

**Table 2:** Univariate and multivariate analyses of variables correlated to biochemical recurrence survival

Variable	Univariate	Multivariate analysis		
	P value	HR	95%CI	P value
Age	0.008	1.387	0.363–5.302	0.632
Prostate volume	0.001	1.964	0.721–5.351	0.187
PSA	< 0.001	1.200	0.385–3.734	0.753
Gleason score	< 0.001	2.487	1.062–5.824	0.036
pT stage	< 0.001	3.654	1.039–12.848	0.043
circKIF18B_003	< 0.001	10.465	1.139–96.190	0.038

### Influence of circKIF18B_003 on proliferation, migration, and invasion characteristics of PCa cells

**Fig. 2:**
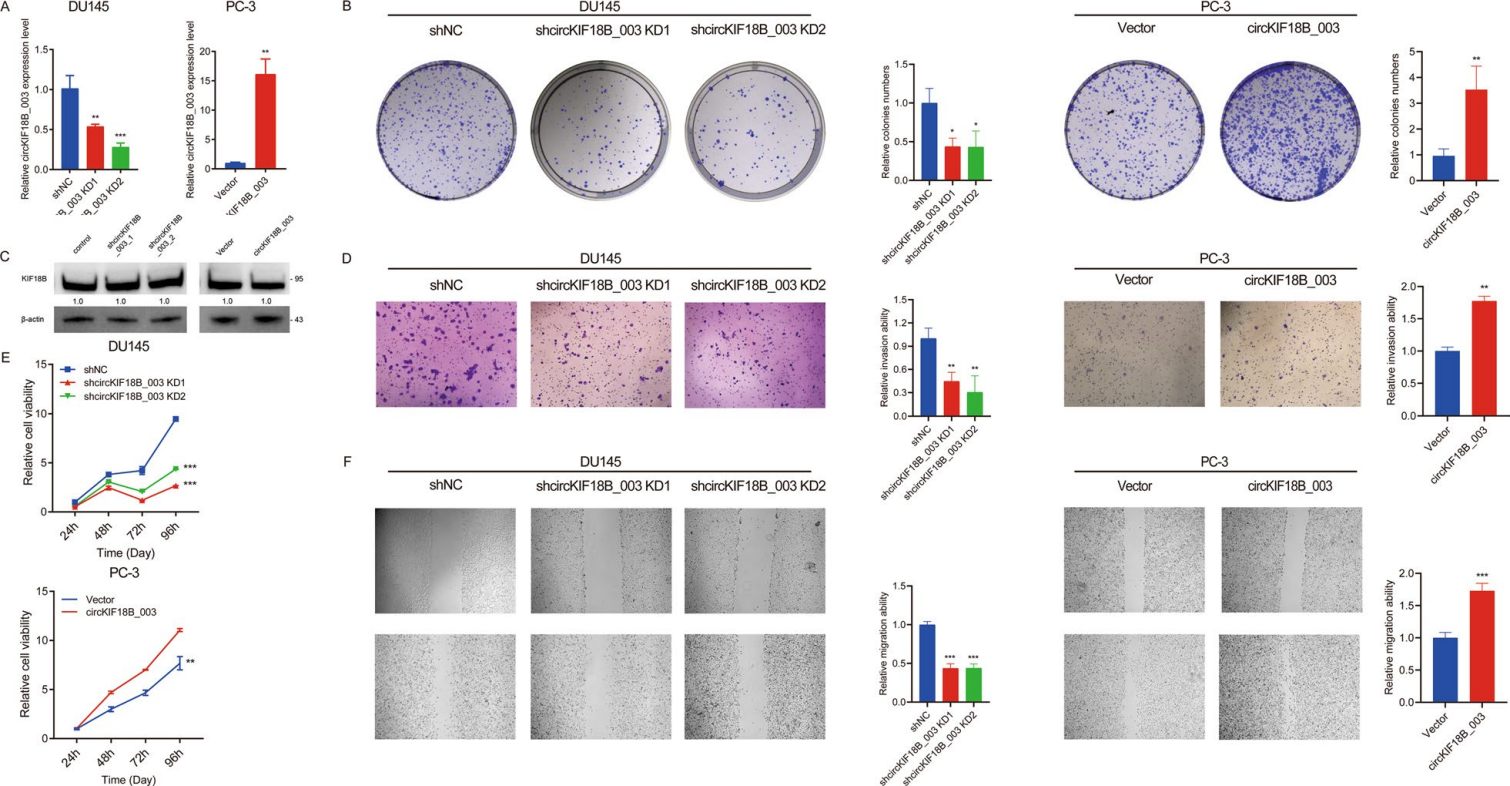
Effects of circKIF18B_003 on PCa Cell Proliferation, Invasion, and Migration. **A** The relative expression levels of circKIF18B_003 in DU145 and PC-3 cell lines post-transfection. DU145 cells were transfected with two shRNAs targeting circKIF18B_003 (for knockdown), while PC-3 cells were transfected with a plasmid promoting circKIF18B_003 overexpression. The graph shows a marked decrease in circKIF18B_003 expression in the DU145 cell line and a significant increase in the PC-3 cell line. **B** Colony formation assay results comparing the effects of circKIF18B_003 downregulation and upregulation on PCa cell viability. Downregulation of circKIF18B_003 led to a decrease in colony formation (indicating reduced cell viability), while upregulation had the opposite effect. **C** KIF18B protein expression levels in DU145 and PC-3 cells after transfection, showing that changes in circKIF18B_003 levels did not influence KIF18B expression. **D** Results from the Transwell invasion assay, demonstrating the impact of circKIF18B_003 modulation on PCa cell invasion capabilities. CircKIF18B_003 knockdown reduced cell invasion, while overexpression led to increased cell invasion. **E** CCK-8 assay results, further demonstrating the impact of circKIF18B_003 modulation on PCa cell viability. Consistent with the colony formation assay, circKIF18B_003 downregulation led to decreased cell viability, while its upregulation promoted cell viability. **F** Results from the wound-healing migration assay, illustrating the influence of circKIF18B_003 on PCa cell migration. Reduced cell migration was observed following circKIF18B_003 knockdown, while overexpression resulted in enhanced cell migration. Unpaired student’s test, Mann–Whitney U test, Kruskal–Wallis test and one-way ANOVA test were used for the statistical analyses. ^*^P < 0.05; ^**^P < 0.01; ^***^P < 0.001

We observed differential expression of circKIF18B_003 in DU145 and PC-3 cell lines, with a higher concentration seen in the DU145 cell line and a lower concentration in the PC-3 cell line. This led us to select the DU145 cell line for knockdown experiments, and the PC-3 cell line for overexpression studies. Remarkably, post-transfection with two shRNAs specifically targeting the back-splice junction site of circKIF18B_003, we noted a significant reduction in circKIF18B_003 expression. On the other hand, cells transfected with a plasmid engineered to promote circKIF18B_003 overexpression exhibited a marked escalation in expression levels (Fig. 2A). Through a colony formation assay, we were able to illustrate the effect of circKIF18B_003 on PCa cell viability. Here, we found that downregulation of circKIF18B_003 compromised PCa cell viability, whereas its upregulation had a promotive effect (Fig. 2B). It is worth noting that although circular RNAs can sometimes influence the production of their host gene's mRNA and consequently reduce the gene's expression, this is not always the case. It is feasible for circular RNAs and their host genes to be regulated independently. This implies that alterations in circKIF18B_003 levels do not necessarily induce changes in KIF18B protein expression or function. Consistent with this, our data indicated that the manipulation of circKIF18B_003 levels did not affect KIF18B expression (Fig. 2C). We further assessed the impact of circKIF18B_003 on PCa cell invasion ability using a Transwell invasion assay. The results showed that circKIF18B_003 knockdown led to decreased invasion ability, while overexpression resulted in enhanced invasion capacity (Fig. 2D). To confirm the effects on cell viability, we conducted CCK-8 assays. These assays provided additional evidence that downregulation of circKIF18B_003 hampered PCa cell viability, whereas upregulation fostered it (Fig. 2E). Finally, we evaluated the impact of circKIF18B_003 on the migratory capacity of PCa cells using wound-healing migration assays. We observed that knockdown of circKIF18B_003 resulted in decreased migration ability, whereas overexpression led to increased migration (Fig. 2F). Through this comprehensive series of assays, we have elucidated the multiple influences of circKIF18B_003 on PCa cell behavior, providing important insights into its role in disease progression.

### CircKIF18B_003 acts as a miR-370-3p sponge in PCa cells

**Fig. 3:**
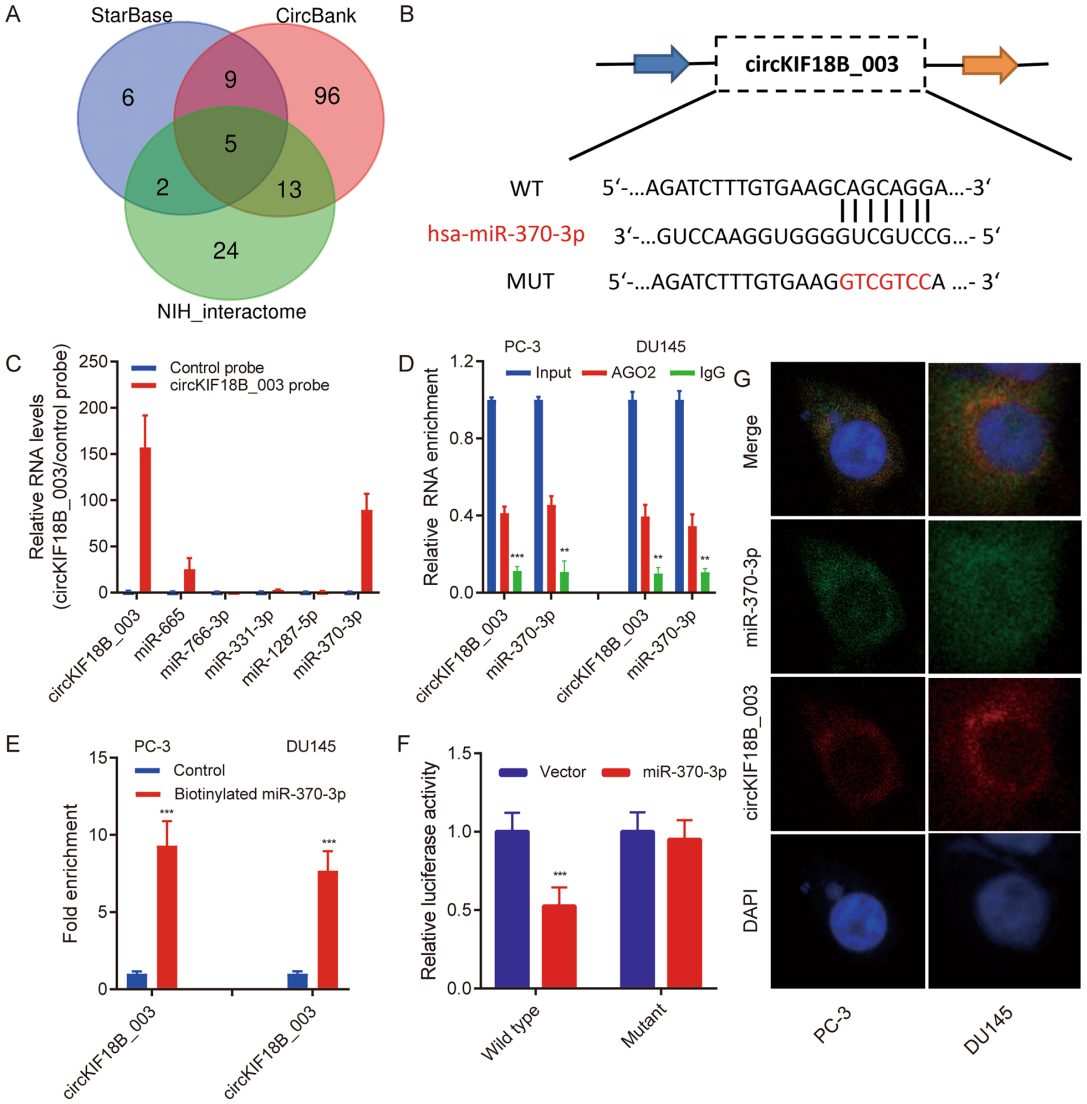
circKIF18B_003 acts as a sponge of miR-370-3p. **A** Schematic illustration exhibiting the overlapping circKIF18B_003 target miRNAs predicted by StarBase, CircBank, and NIH_interactome databases. **B** Putative binding site of miR-370-ep in circKIF18B_003 was predicted using StarBase version 3.0. **C** circKIF18B_003 and corresponding negative control probes were used in circRIP assays in circKIF18B_003-overexpression PC-3 cells. **D** RIP assays were performed using an antibody against AGO2 on extracts from PC-3 and DU145 cells. E. circKIF18B_003 level in the streptavidin captured fractions from the PCa cell lysates after transfection with biotinylated miR-370-3p or negative control. **F** The luciferase activity of pLG3-circKIF18B_003 in HEK-293 T cells after co-transfection with miR-370-3p mimics. **G** circKIF18B_003 and miR-370-3p were evaluated in the indicated cell lines by FISH assay. ^***^P < 0.001; ns, not significant

Using StarBase, CircBank, and NIH_Interactome databases, potential miRNAs targeted by circKIF18B_003 were predicted. Only five miRNAs (miR-665, miR-331-3p, miR-766-3p, miR-1287-5p, and miR-370-3p) were common across all three databases (Fig. 3A). The putative binding site of miR-370-3p in circKIF18B_003 was identified using StarBase version 3.0 (Fig. 3B). A circKIF18B_003 specific probe was designed to perform a circRIP assay, investigating interactions between circKIF18B_003 and the identified miRNAs in circKIF18B_003-overexpressing PC-3 cells. Results indicated an interaction between miR-370-3p and circKIF18B_003, as miR-370-3p was enriched (Fig. 3C). RIP assay with an Argonaute 2 (AGO2) antibody in DU145 and PC-3 cells revealed both circKIF18B_003 and miR-370-3p enrichment, suggesting circKIF18B_003 may serve as a miR-370-3p binding platform (Fig. 3D). This interaction was further confirmed through an RIP assay using a biotinylated miR-370-3p probe, which effectively captured circKIF18B_003 (Fig. 3E). To validate the circKIF18B_003 and miR-370-3p interaction in HEK-293 T cells, luciferase reporter experiments were performed. Transfection with miR-370-3p mimics significantly decreased luciferase activity driven by the wild-type reporter, while activity driven by the mutant reporter remained unaffected (Fig. 3F). FISH assays revealed both circKIF18B_003 and miR-370-3p localized in the cytoplasm of DU145 and PC-3 cells (Fig. 3G). These collective findings confirm the binding of circKIF18B_003 to miR-370-3p in PCa cells.

### circKIF18B_003 upregulates ACACA level by miR-370-3p sponging

**Fig. 4:**
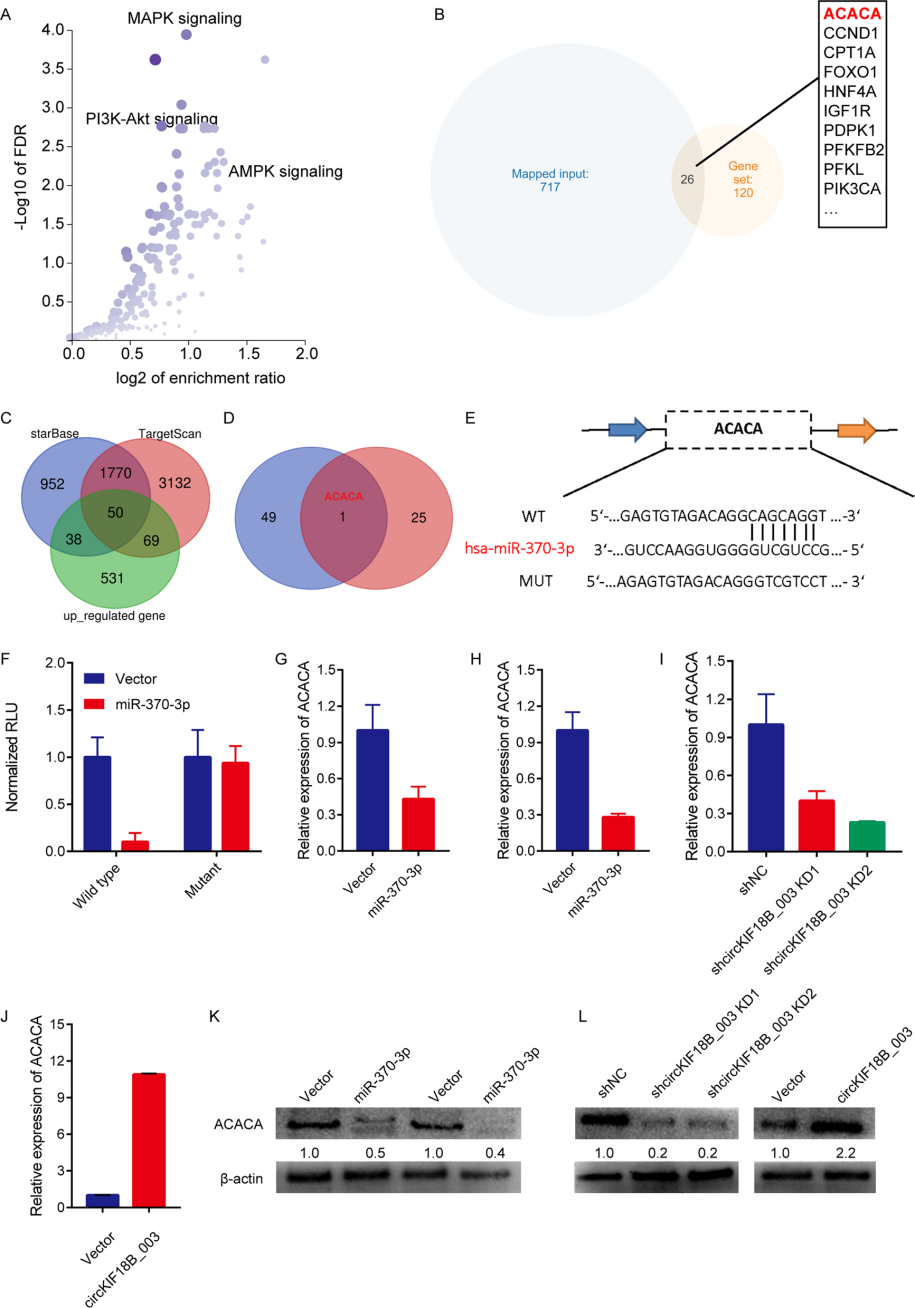
circKIF18B_003 upregulates ACACA by sponging miR-370-3p. **A** KEGG analyses of circKIF18B_003-targeted mRNAs. The main signaling pathways, including MAPK signaling, PI3K-AKT signaling, and AMPK signaling, are shown in the bubble chart. **B** Diagram illustrating the overlap between circKIF18B 003-targeted mRNAs and mRNAs in the AMPK signaling pathway. **C** Schematic depicting the overlapping potential targets of miR-370-3p predicted by StarBase and CircBank databases and upregulated mRNAs differentially expressed between PCa tissues and corresponding normal tissues. **D** Schematic illustration exhibiting the overlapping screened miR-370-3p targeted genes and mRNAs in the AMPK signaling pathway. **E** Schematic illustration exhibiting the putative binding site of miR-370-3p in ACACA mRNA predicted by StarBase version 3.0. **F** The luciferase activity of GP-miRGLO-ACACA in HEK-293T cells after co-transfection with miR-370-3p mimics. **G**, **H** Relative ACACA mRNA expression in DU145 and PC-3 PCa cell lines. **I** Relative ACACA mRNA expression in DU145 cells after knockdown of circKIF18B_003. **J** Relative ACACA mRNA expression in PC-3 PCa cell line after overexpression of circKIF18B_003. **K** Relative ACACA protein expression in DU145 and PC-3 PCa cell lines transfected with miR-370-3p mimics. **L** Relative ACACA protein expression in DU145 and PC-3 cell lines after knocking down and overexpressing the expression of circKIF18B_003

Using the StarBase and CircBank databases, we identified ACACA as the likely mRNA target of miR-370-3p. KEGG analysis revealed enrichment of the identified mRNAs in insulin resistance, AMPK, and MAPK signaling pathways (Fig. 4A). Notably, ACACA was a common factor among the miR-370-3p targets, upregulated mRNAs, and AMPK pathway–enriched genes (Fig. 4B–D). We found the putative miR-370-3p binding site in ACACA mRNA using StarBase version 3.0 (Fig. 4E). We hypothesized that circKIF18B_003 promotes malignant PCa cell growth by reducing miR-370-3p, thus upregulating ACACA. Transfection with miR-370-3p mimics significantly decreased luciferase activity in cells containing wild-type ACACA, relative to those with mutant ACACA (Fig. 4F). Overexpressing miR-370-3p attenuated ACACA mRNA expression in DU145 and PC-3 cells (Fig. 4G and 4H), while downregulating circKIF18B_003 decreased ACACA mRNA expression in DU145 cells and upregulated it in PC-3 cells (Fig. 4I and J). ACACA protein expression followed a similar pattern (Fig. 4K and L). These findings imply that circKIF18B_003 upregulates ACACA expression by sponging miR-370-3p.

### ACACA downregulation inhibits PCa cell proliferation, migration, and invasion

**Fig. 5:**
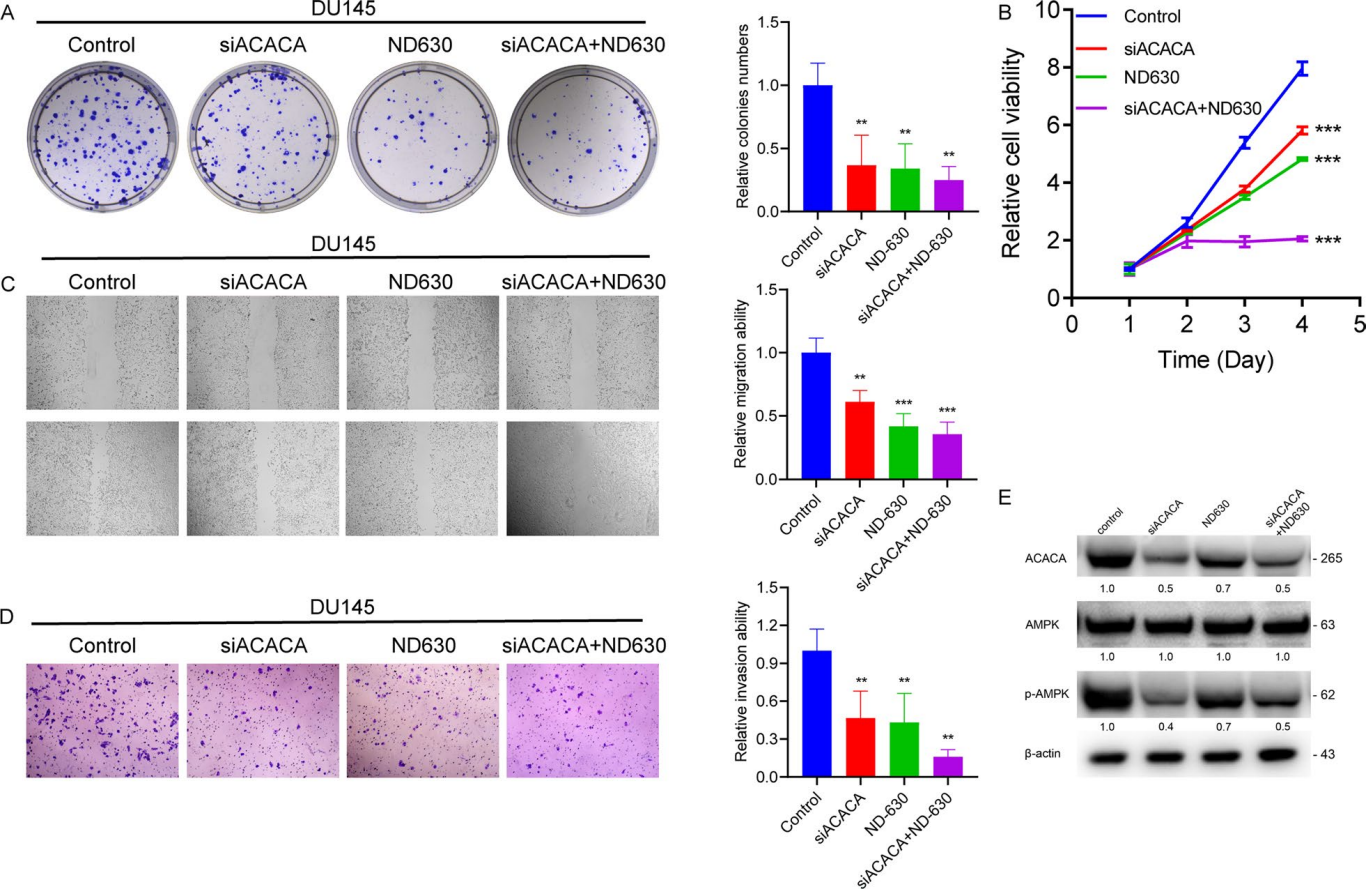
ACACA and its inhibitor ND630 regulate PCa cell activity in vitro. **A**–**D** CCK8, Colony formation, wound healing, and Transwell invasion assays were performed in DU145 cells treated with control, siACACA, ND630, and siACACA + ND630. **E** Western blot assay was used to determine the ACACA and p-AMPK levels in DU145 PCa cells with different treatments. β-actin was used as a loading control. One-way ANOVA test and Kruskal–Wallis test were used for the statistical analyses. ^**^P < 0.01, ^***^P < 0.001

We assessed the effects of ACACA knockdown on PCa cell behavior. ACACA knockdown markedly reduced cell viability, migration, and invasion in PCa cells (Fig. 5A–D). Similar effects were observed using ND-630, an ACACA inhibitor (Fig. 5A–D). ACACA knockdown decreased phospho-AMPKα (Thr172), indicating AMPK pathway activation (Fig. 5E). In the PC-3 cell line, as depicted in Additional file 1: Fig. S1, downregulation of ACACA by siACACA or its inhibition by ND630 significantly reduced cell viability, migration, and invasion. These findings further confirm the oncogenic role of ACACA in PCa cells.

### ACACA downregulation reverses malignancy induced by circKIF18B_003

**Fig. 6:**
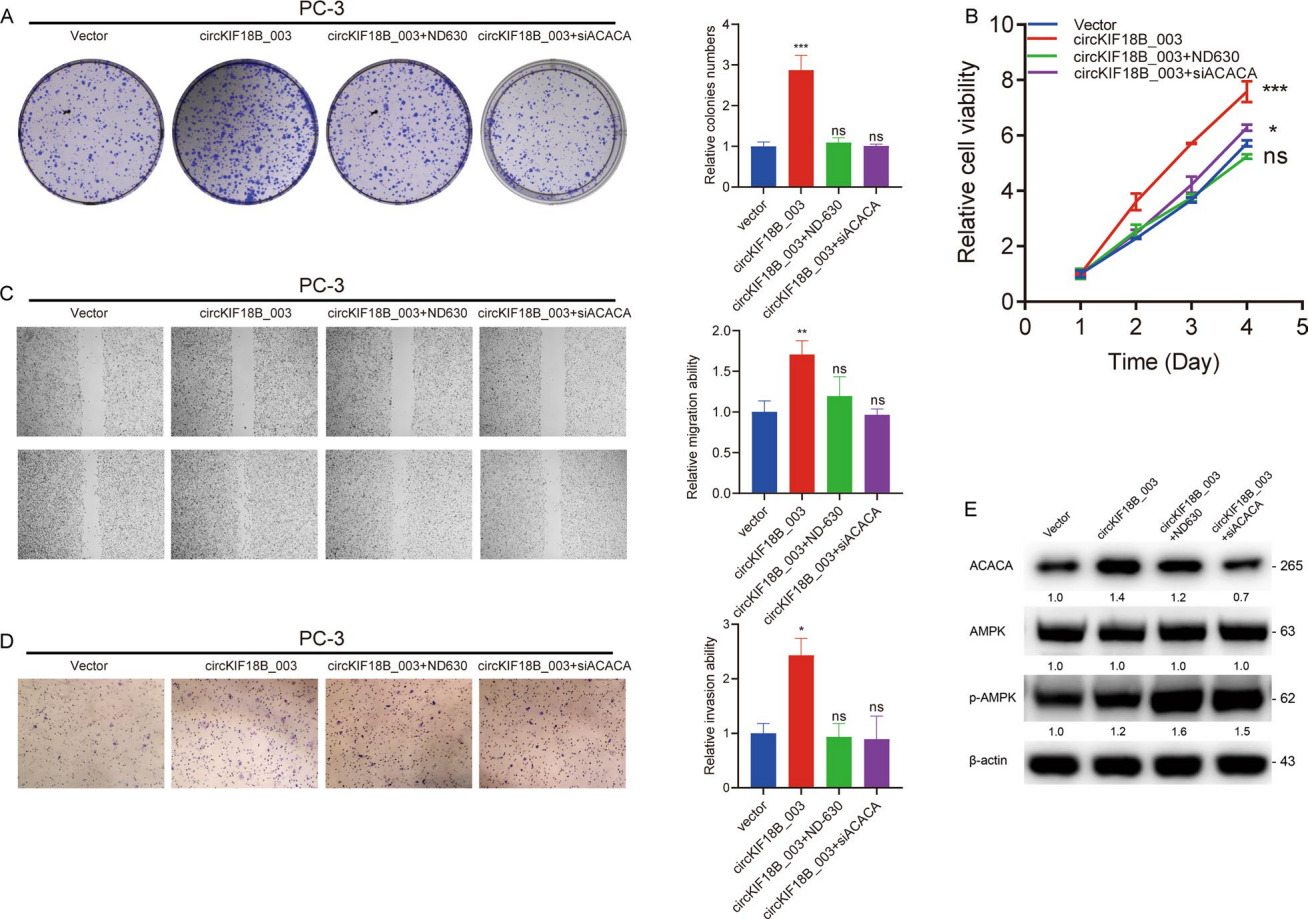
Knockdown of ACACA attenuates PCa progression and reverses circKIF18B_003-induced malignant phenotypes in PCa cell lines. **A**–**D** Colony formation, CCK8, wound healing, and Transwell invasion assays were performed in PC-3 cells treated with vector, circKIF18B_003, circKIF18B_003 + ND630, and circKIF18B_003 + siACACA. **E** Western blot assay was used to determine the ACACA and p-AMPK protein expression levels in PC-3 cells treated as indicated. β-actin was used as a loading control. One-way ANOVA test and Kruskal–Wallis test were used for the statistical analyses. ^*^P < 0.05, ^**^P < 0.01, ^***^P < 0.001, ns: no significant difference

Rescue experiments showed that inhibiting ACACA, either genetically or pharmacologically, hindered the proliferation, migration, and invasion induced by overexpressed circKIF18B_003 (Fig. 6A–D), suggesting that ACACA downregulation can reverse the malignant phenotype induced by circKIF18B_003. However, upon overexpression of circKIF18B_003, we did not observe a significant change in the phosphorylation status of AMPKα at Thr172 (Fig. 6E). In the DU145 cell line, as shown in Additional file 2: Fig. S2, overexpression of circKIF18B_003 significantly promoted cell proliferation, invasion, and migration. However, the introduction of ND630 or siACACA effectively negated these effects of circKIF18B_003, suggesting that ACACA plays a crucial role in circKIF18B_003 induced proliferation, migration, and invasion. These findings provide additional support to the central role of ACACA in mediating the effects of circKIF18B_003.

### circKIF18B_003 and ACACA regulate lipid metabolism

**Fig. 7:**
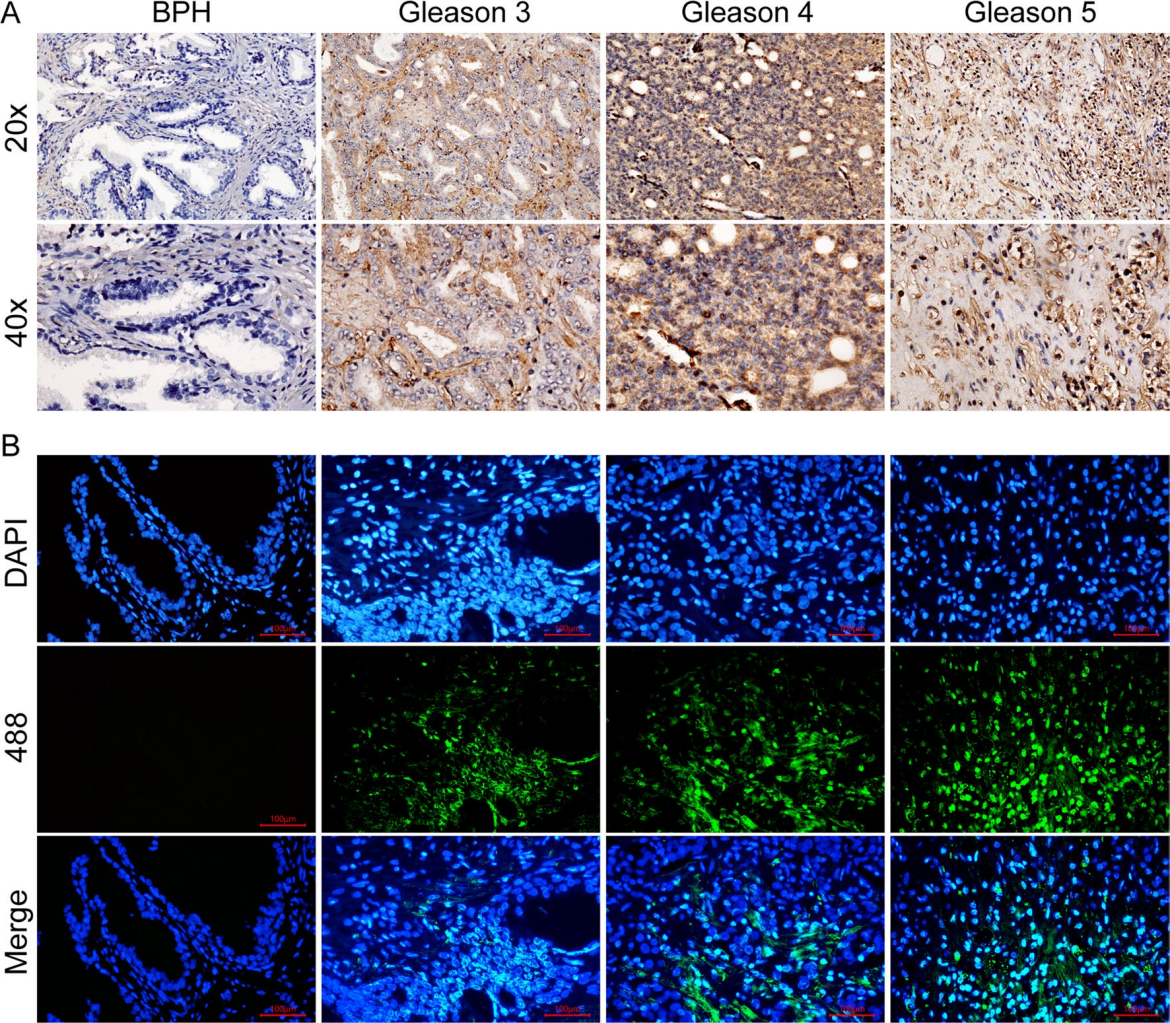
ACACA Expression in Prostate Cancer (PCa) and Benign Prostatic Hyperplasia (BPH) Tissues. **A** Immunohistochemistry assay was utilized to detect ACACA expression in PCa and BPH tissues. The stained tissue samples were observed and imaged under a light microscope. **B** Immunofluorescence assay was performed to further validate ACACA expression in PCa and BPH tissues. Images captured under a fluorescence microscope were used for comparative analysis of ACACA expression between PCa and BPH tissues

**Fig. 8:**
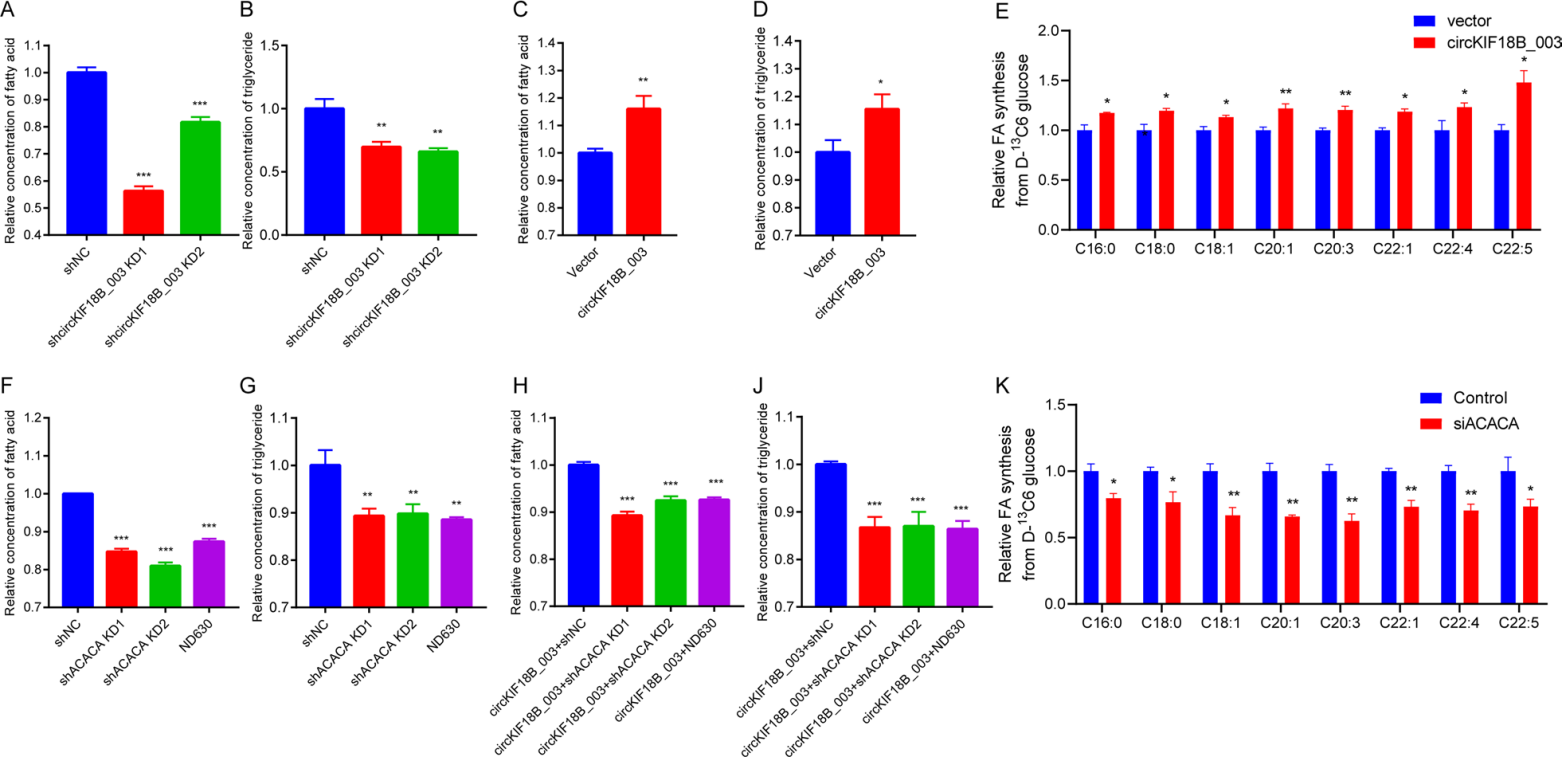
CircKIF18B_003 and ACACA Regulate Lipid Metabolism in Prostate Cancer Cells. **A**–**D** The formation of fatty acids (FA) and triglycerides (TG) in prostate cancer cells is regulated by circKIF18B_003. In DU145 cells, knockdown of circKIF18B_003 decreases the levels of these lipids, while in PC-3 cells, overexpression of circKIF18B_003 increases their levels. **E** Overexpression of circKIF18B_003 in PC-3 cells greatly enhances the incorporation of glucose into FA, as evidenced by liquid chromatography-mass spectrometry (LC–MS) analysis. **F**–**J** The glucose-incorporating effect of circKIF18B_003 overexpression is reversed by either knockdown of ACACA or treatment with ND630, suggesting a regulatory role of ACACA in lipid synthesis.** K** Knockdown or inhibition of ACACA by siACACA in DU145 cells significantly decreases the incorporation of glucose into de novo FA synthesis. These results collectively suggest a pivotal role of circKIF18B_003 and ACACA in enhancing glucose utilization for FA synthesis in prostate cancer cells, thereby contributing to the malignant phenotype of these cells. *P < 0.05, **P < 0.01, ***P < 0.001

ACACA expression in PCa samples was found to increase with Gleason scores, but was low or absent in benign prostate hyperplasia (BPH) samples (Fig. 7A and B). ELISA showed that circKIF18B_003 knockdown decreased fatty acid and triglyceride levels in DU145 cells, while circKIF18B_003 overexpression increased these levels in PC-3 cells (Fig. 8A–D). To further elucidate the role of circKIF18B_003 and ACACA in lipid metabolism, we conducted a liquid chromatography-mass spectrometry (LC–MS) analysis to measure glucose incorporation into de novo fatty acid synthesis in PC-3 cells. We found that the overexpression of circKIF18B_003 significantly increased the incorporation of glucose into fatty acids in PC-3 cells (Fig. 8E). These effects were reversed by ACACA knockdown or ND630 treatment (Fig. 8F–J), suggesting that ACACA may regulate lipid synthesis. Similarly, when ACACA was knocked down or inhibited by siACACA, there was a significant decrease in the incorporation of glucose into de novo fatty acid synthesis in DU145 (Fig. 8K). These results indicate that both circKIF18B_003 and ACACA greatly enhance glucose utilization for fatty acid synthesis in PCa cells. In summary, our LC–MS results provide strong evidence that circKIF18B_003 and ACACA play a pivotal role in fatty acid synthesis, which is a key feature of cancer cell metabolism and may contribute to the malignant phenotype of PCa cells.

### Metabolic impact of ACACA downregulation

Our investigation into the metabolic effects of ACACA downregulation and circKIF18B_003 overexpression, examined through Seahorse XF24 assays, revealed significant alterations in cellular metabolism across both DU145 and PC-3 cell lines (Additional file 3: Fig. S3A–L). In the DU145 cell line (Additional file 3: Fig. S3A–F), cells were categorized into Control and siACACA groups. Notably, ACACA downregulation resulted in substantial changes in the oxygen consumption rate (OCR), as portrayed in Additional file 3: Fig. S3A. Treatment with metabolic inhibitors—Oligomycin (OLI), FCCP, and Rotenone/Antimycin A (Rtn/AA)—unveiled a unique OCR pattern in the siACACA group compared to the control, indicating a distinct metabolic shift.

This shift was further underscored by a significant reduction in basal respiration (Additional file 3: Fig. S3B) and maximal respiration (Additional file 3: Fig. S3C) in the siACACA group. ATP production (Additional file 3: Fig. S3D) mirrored these changes, showing a notable decrease in comparison to the control. Interestingly, the proton leak (Additional file 3: Fig. S3E) did not display a significant difference between the two groups. However, the spare respiratory capacity (Additional file 3: Fig. S3F), another key aspect of cellular metabolism, was reduced in the siACACA group, further confirming the metabolic impact of ACACA downregulation.

### Metabolic impact of circKIF18B_003 overexpression

Turning our attention to the PC-3 cell line (Additional file 3: Fig. S3G–L), we divided the cells into Vector and circKIF18B_003 groups. Upon overexpression of circKIF18B_003, we observed a series of significant metabolic changes. The OCR, depicted in Additional file 3: Fig. S3G, showed a different pattern in the circKIF18B_003 group compared to the vector control following the sequential addition of OLI, FCCP, and Rtn/AA.

Further analysis revealed a significant increase in basal respiration (Additional file 3: Fig. S3H), maximal respiration (Additional file 3: Fig. S3I), and ATP production (Additional file 3: Fig. S3J) in circKIF18B_003 overexpressing cells. Despite these changes, the proton leak (Additional file 3: Fig. S3K) remained relatively consistent. However, the spare respiratory capacity (Additional file 3: Fig. S3L) was markedly elevated, indicating a higher metabolic potential in circKIF18B_003 overexpressing cells.

Collectively, these findings imply that both ACACA downregulation and circKIF18B_003 overexpression play significant roles in reprogramming cellular metabolism. Such metabolic shifts could be a contributing factor to the observed changes in cell proliferation, migration, and invasion, thus underscoring the importance of these genetic alterations in cancer cell biology.

### In vivo efficacy of ND630, siACACA, and shcircKIF18B_003 in prostate cancer

**Fig. 9:**
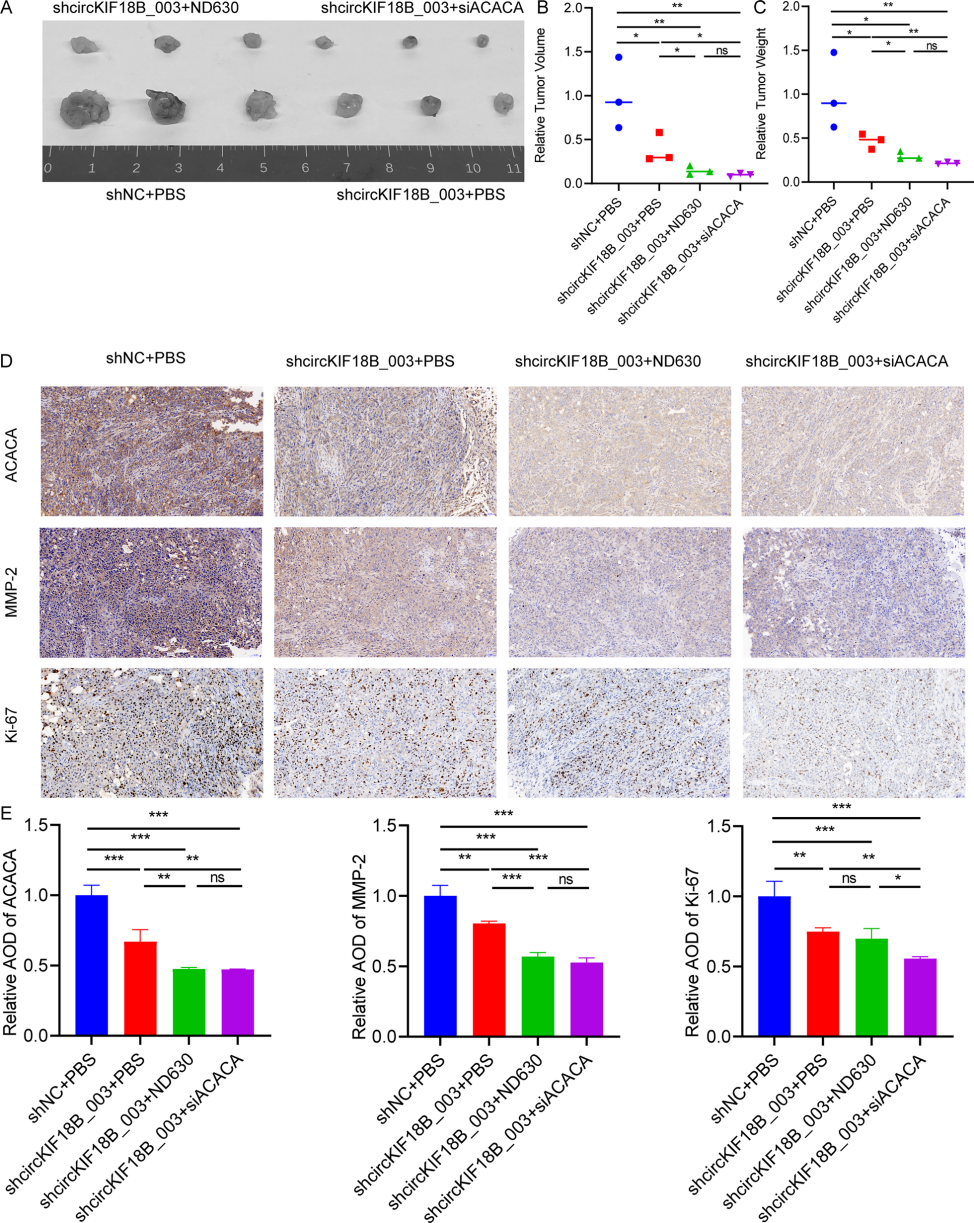
The efficacy of ND630 and siACACA in PCa in vivo. **A** Images of tumors derived from PC-3 shNC, PC-3-shcircKIF18B_003 or PC-3-shcircKIF18B_003 + siACACA cells in animals treated with PBS or ND630. **B**, **C** Average tumor volume and weight from animals injected with PC-3 shNC, PC-3-shcircKIF18B_003 or PC-3-shcircKIF18B_003 + siACACA cells and treated with PBS or ND630. **D** IHC assay were used to determine the expression of ACACA, Ki67, and MMP2 in tumors. **E** Quantification of mean optical density of the protein staining of ACACA, Ki67, and MMP2

Our results demonstrate the impact of ND630 and siACACA and shcircKIF18B_003 on PCa in vivo. In Fig. 9A, images of tumors originating from PC-3 shNC, PC-3-shcircKIF18B_003, or PC-3-shcircKIF18B_003 + siACACA cells in mice treated with PBS or ND630 visually display the inhibitory effect of both ND630 and siACACA on tumor growth. Following this, Fig. 9B and C present the average tumor volume and weight, respectively, from animals injected with the different cell types and treated with either PBS or ND630. These data highlight a significant reduction in both tumor volume and weight in the shcircKIF18B_003 + siACACA and shcircKIF18B_003 + ND630 groups compared to the shNC + PBS control group. The immunohistochemistry (IHC) assay results in Fig. 9D show the expression of ACACA, Ki67, and MMP2 in tumors derived from the different cell types, revealing a decrease in the expression of these proteins in the shcircKIF18B_003 + siACACA and shcircKIF18B_003 + ND630 groups compared to the control group. Finally, the quantification of the mean optical density of the protein staining of ACACA, Ki67, and MMP2 in Fig. 9E corroborates the IHC results, showing a significant reduction in the expression of these proteins in the shcircKIF18B_003 + siACACA and shcircKIF18B_003 + ND630 groups compared to the control group. Taken together, these findings demonstrate the inhibitory effects of ND630 and siACACA on the growth of PCa in vivo by reducing tumor volume and weight and downregulating the expression of ACACA, Ki67, and MMP2.

## Discussion

The prognosis of prostate cancer (PCa) patients can be improved through early diagnosis. Although the utilization of prostate-specific antigen (PSA) test–initiated prostate biopsy has led to an increase in the rate of early diagnosis, it has also resulted in an uptick in unnecessary biopsies and associated complications [28]. Currently, PCa metastases are a primary cause of patient mortality, which significantly shortens their lifespan. While localized PCa can be addressed with radical prostatectomy, treatment options for advanced metastatic PCa are limited and generally ineffective. Although localized PCa be treated by radical prostatectomy, the treatment strategies for advanced metastatic PCa is limited and ineffective. Consequently, it is crucial to identify novel and effective therapeutic targets to improve PCa clinical outcomes.

Consequently, it is crucial to identify novel and effective therapeutic targets to improve PCa clinical outcome. Existing research has established connections between circRNAs and various physiological and pathological processes. Some studies, such as those by Ding et al. [29] and Gao et al. [30], suggest that the dysregulation of certain circRNAs may influence PCa progression and drug sensitivity. However, the role of circRNAs in PCa still requires more comprehensive investigation. We previously reported the role of KIF18B in the proliferation and invasion of PCa in vitro and in vivo [16]. Overexpression of KIF18B is associated with poor cancer free survival and non-biochemical recurrence survival in PCa. Wu et al. [31] discovered that KIF18B enhances cervical cancer tumor growth by activating the Wnt/-catenin pathway. Gao et al. [32] revealed that KIF18B stimulates tumor progression in osteosarcoma. Ji et al. [33] demonstrated that KIF18B works as a regulator of microtubule mobility to expedite lung adenocarcinoma tumor growth. Therefore, we speculated that KIF18B derived circRNA, circKIF18B_003, may have a crucial function in the development of PCa. We found that circKIF18B_003 was overexpressed in PCa tissues. High expression of circKIF18B_003 was confirmed in clinical samples and was associated with a poor outcome of PCa patients. Our results show that circKIF18B_003 may induce PCa progression and reshape lipid metabolism reprogramming.

CircRNAs can function as miRNA sponges to downregulate the inhibitory effect of miRNA on its target genes [34]. For instance, Ma et al. [35] revealed that circ_0004872 acts as a sponge for miR-224 to regulate p21 and Smad4 to affect gastric cancer progression. Xue et al. [36] revealed that circ-AKT3 suppresses tumor metastasis by upregulating E-cadherin expression by binding miR-296-3p competitively in clear cell renal cell carcinoma. Our RIP assay, luciferase, and western blot results confirmed that circKIF18B_003 interacts with miR-370-3p and functions as a sponge for it. These results demonstrate that circKIF18B_003 works as a competitive endogenous RNA to influence PCa progression via the miR-370-3p/ACACA axis.

Extensive evidence has shown that overexpression of ACACA is essential for proliferation and invasion and promoting glucose-mediated fatty acid synthesis in many human cancers. For example, Ye et al. [37] showed that ACACA is upregulated in liver cancers and increases malignancies of liver cancer cells. Wang et al. [12] reported that lncRNA CTD-2245E15.3 support the growth of non-small cell lung cancer through promoted ACACA. Guo et al. [38] demonstrated that lncRNA HNRNPA2B1 promotes esophageal cancer progression by upregulating ACACA. The results demonstrated that inhibition of ACACA could suppress fatty acid synthesis and tumor growth. ACACA is involved in many cancer metabolism reprogramming processes, as cancer cells depend on fatty acids for membrane synthesis. Lipid signaling plays a crucial function in cell signaling transduction, which is essential for the biochemical processes of cancer cells. Fatty acids act as an energy resource for cancer cell metabolism. Prior studies have reported that pharmacologic inhibition of ACACA decreased the mortality of patients with metabolic syndrome via limiting fatty acid synthesis in lipogenic tissues. Luo et al. [21] revealed that ACACA rewired cancer metabolism, allowing cancer cells to withstand the Warburg effect suppression by cetuximab. Rios et al. [39] provided evidence that ACACA plays a key part in breast cancer metastasis and recurrence by regulating ACACA-dependent protein acetylation. Nishi et al. [40] stated that the use of a combination of inhibitors of ACACA might become a potential therapeutic target for the treatment of pancreatic cancer. In this work, we demonstrated that circKIF18B_003 functions as a miR-370-3p sponge to attenuate ACACA inhibition, therefore altering lipid metabolism reprogramming. The ND-630 or siACACA significantly suppressed the proliferation of PCa cells as compared to single treatments or the control group.

Our study elucidates the pivotal role of ACACA and circKIF18B_003 in governing lipid metabolism and overall cellular metabolism in prostate cancer cells. Both these factors appear to function in opposing directions, with ACACA downregulation and circKIF18B_003 overexpression leading to contrasting alterations in cellular metabolism. ACACA is a key enzyme involved in the initial step of fatty acid synthesis. Consequently, its downregulation can profoundly influence lipid metabolism. In our experiments, downregulation of ACACA resulted in a unique pattern of OCR, implying a potential shift in energy metabolism possibly due to diminished fatty acid synthesis. This shift may force cells to resort to alternative energy pathways, such as glycolysis or amino acid catabolism, reflective of metabolic flexibility where cells adapt to the availability of substrates and specific energetic demands. Furthermore, the effects of ACACA downregulation may extend beyond fatty acid synthesis. Alterations in lipid metabolism could also impact cell membrane composition and fluidity, intracellular signaling pathways, and generation of lipid-derived signaling molecules. Therefore, the observed effects on OCR and overall cellular metabolism could be a result of a complex interplay of direct and indirect influences stemming from ACACA downregulation.

In contrast, circKIF18B_003 overexpression led to a series of significant metabolic changes in the PC-3 cell line. Notably, we observed an increase in OCR, basal respiration, maximal respiration, ATP production, and spare respiratory capacity. These alterations suggest a shift towards enhanced oxidative phosphorylation, potentially fueled by increased fatty acid synthesis driven by circKIF18B_003 overexpression. Despite the contrasting impacts of ACACA downregulation and circKIF18B_003 overexpression, both appear to play a crucial role in metabolic reprogramming in prostate cancer cells. These reprogrammed metabolic pathways potentially contribute to changes in cell proliferation, migration, and invasion, underlining the relevance of these genetic alterations in cancer cell biology.

Finally, we acknowledge the limitations of our study, particularly in categories such as PSA > 10, Gleason score > 7, and pT stage T3-T4, where the number of patients with low expression of circKIF18B_003 was relatively small. This limited sample size might affect the validity and reliability of our statistical analysis, and the results should be interpreted with caution.

## Conclusion

Our study demonstrated that circKIF18B_003 could serve as a novel biomarker and potential therapeutic target for PCa. Further studies are required to validate our findings and to explore the precise molecular mechanisms underlying the influence of circKIF18B_003 on PCa progression. Our study provides a new perspective on the role of circRNAs in PCa and adds to the growing body of evidence suggesting the potential use of circRNAs as biomarkers and therapeutic targets in human cancers.

### Supplementary Information

**Additional file 1: Figure S1.** Additional Functional Tests of siACACA and ND630 in PC-3 Cell Line. A: Colony formation assay results showing that all treatment groups (siACACA, ND630, and siACACA + ND630) displayed a significant decrease in colony formation compared to the control group, indicating reduced cell viability. B: Wound-healing migration assay results illustrating that all treatment groups (siACACA, ND630, and siACACA + ND630) showed a considerable reduction in cell migration compared to the control group. C: Results from the Transwell invasion assay demonstrating that all treatment groups (siACACA, ND630, and siACACA + ND630) experienced a significant decline in cell invasion compared to the control group. D: Combined statistical graphs for colony formation assay, wound-healing migration assay, and transwell invasion assay results and CCK-8 assays, further highlighting the significant decrease in cell viability in all treatment groups (siACACA, ND630, and siACACA + ND630) compared to the control group. For statistical analyses, unpaired student’s test, Mann–Whitney U test, Kruskal–Wallis test, and one-way ANOVA test were utilized. *P < 0.05; **P < 0.01; ***P < 0.001.

**Additional file 2: Figure S2.** Influence of circKIF18B_003 on Cell Proliferation, Invasion, and Migration in DU145 Cell Line. A: Colony formation assay results showing that the circKIF18B_003 overexpression group showed the most significant increase in colony formation compared to the vector control group, thus indicating enhanced cell viability. The groups with circKIF18B_003 + ND630 and circKIF18B_003 + siACACA showed no significant changes in colony formation. B: Wound-healing migration assay results illustrating that overexpression of circKIF18B_003 resulted in the most noticeable increase in cell migration compared to the vector control group. The groups with circKIF18B_003 + ND630 and circKIF18B_003 + siACACA did not exhibit significant changes in cell migration. C: Transwell invasion assay results demonstrating that the circKIF18B_003 overexpression group had the highest increase in cell invasion compared to the vector control group. The groups treated with circKIF18B_003 + ND630 and circKIF18B_003 + siACACA did not display notable changes in cell invasion. D: Combined statistical graphs for colony formation assay, wound-healing migration assay, and transwell invasion assay results and CCK-8 assays, further confirming the most significant increase in cell viability in the circKIF18B_003 overexpression group compared to the vector control group. The groups with circKIF18B_003 + ND630 and circKIF18B_003 + siACACA did not show significant changes in cell viability. For statistical analyses, unpaired student’s test, Mann–Whitney U test, Kruskal–Wallis test, and one-way ANOVA test were utilized. *P < 0.05; **P < 0.01; ***P < 0.001.

**Additional file 3: Figure S3.** Metabolic changes in DU145 and PC-3 cell lines following ACACA downregulation and circKIF18B_003 overexpression. (A−F) Metabolic changes in the DU145 cell line due to ACACA downregulation. (A) Oxygen consumption rate (OCR) shows substantial alterations following ACACA downregulation. (B) Basal respiration and (C) maximal respiration are both significantly reduced in siACACA group. (D) ATP production is notably decreased in the siACACA group compared to the control. (E) Proton leak shows no significant difference between the two groups. (F) Spare respiratory capacity is reduced in the siACACA group, confirming the metabolic impact of ACACA downregulation. (G–L) Metabolic changes in the PC-3 cell line due to circKIF18B_003 overexpression. (G) OCR reveals a different pattern in the circKIF18B_003 group compared to the control. (H) Basal respiration, (I) maximal respiration, and (J) ATP production all show a significant increase in the circKIF18B_003 group. (K) Proton leak remains relatively consistent across groups. (L) Spare respiratory capacity is markedly elevated in the circKIF18B_003 group, indicating a higher metabolic potential.

**Additional file 4: Table S1.** Sequences of Primers used for RT-qPCR.

**Additional file 5: Table S2.** List of Primary Antibodies Used in the Study.

## Data Availability

All data generated or analyzed during this study are included either in this article or in the Methods, Tables, Figures, and Figure Legends files. The processed data are available from the corresponding author upon reasonable request.

## Abbreviations

PCa: Prostate cancer
circRNAs: Circular RNAs
ACACA: Acetyl-CoA carboxylase alpha
FAS: Fatty acid synthase
KIF18B: Kinesin family member 18B
miR-370-3p: MicroRNA-370-3p
qRT-PCR: Quantitative real-time polymerase chain reaction
shRNAs: Small hairpin RNAs
cDNA: Complementary DNA
siRNA: Small interfering RNA
miRNA: MicroRNA
shNC: Negative control shRNA
IHC: Immunohistochemistry
FISH: Fluorescence in situ hybridization
DAPI: 4',6-Diamidino-2-phenylindole
CCK-8: Cell Counting Kit-8
RIP: RNA immunoprecipitation
Luciferase: Firefly luciferase
Co-IP: Co-immunoprecipitation
KSFM: Keratinocyte Serum-Free Medium
MEM: Minimum Essential Medium
DMEM: Dulbecco's Modified Eagle Medium
PBS: Phosphate-buffered saline
DMSO: Dimethyl sulfoxide
TG: Triglyceride
FA: Fatty acid
SD: Standard deviation
ANOVA: Analysis of variance
SPSS: Statistical Package for the Social Sciences
ELISA: Enzyme-linked immunosorbent assay
BPH: Benign prostate hyperplasia
MMP-2: Matrix metalloproteinase-2
