# Supplementary figures and images for: ND630 controls ACACA and lipid reprogramming in prostate cancer by regulating the expression of circKIF18B_003
Source: J Transl Med. 2023 Dec 4;21:877. doi: 10.1186/s12967-023-04760-w (PMC10694902; doi:10.1186/s12967-023-04760-w)

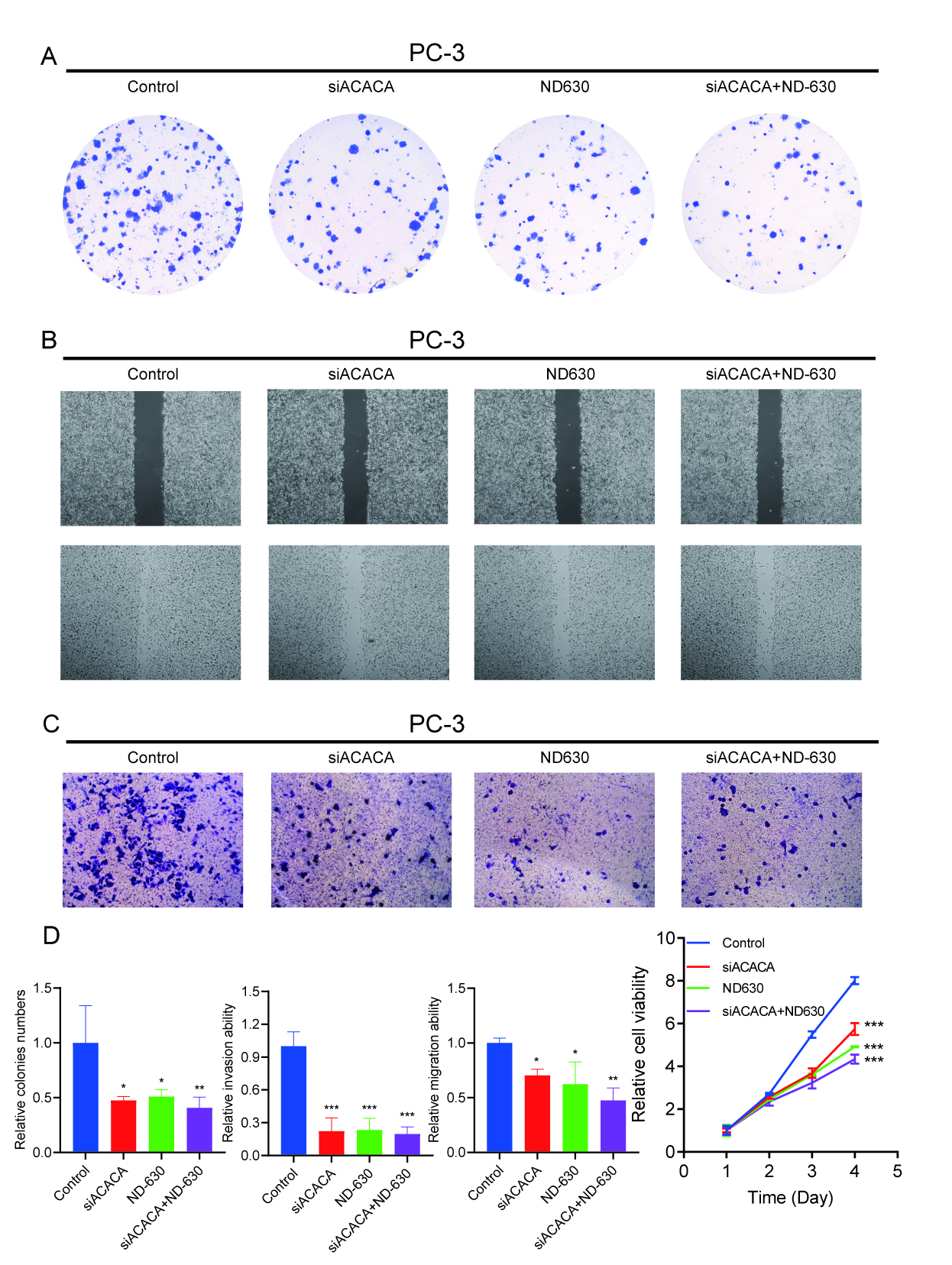

Supplement: Supplementary file 1 — Additional file 1: Figure S1. Additional Functional Tests of siACACA and ND630 in PC-3 Cell Line. A: Colony formation assay results showing that all treatment groups (siACACA, ND630, and siACACA + ND630) displayed a significant decrease in colony formation compared to the control group, indicating reduced cell viability. B: Wound-healing migration assay results illustrating that all treatment groups (siACACA, ND630, and siACACA + ND630) showed a considerable reduction in cell migration compared to the control group. C: Results from the Transwell invasion assay demonstrating that all treatment groups (siACACA, ND630, and siACACA + ND630) experienced a significant decline in cell invasion compared to the control group. D: Combined statistical graphs for colony formation assay, wound-healing migration assay, and transwell invasion assay results and CCK-8 assays, further highlighting the significant decrease in cell viability in all treatment groups (siACACA, ND630, and siACACA + ND630) compared to the control group. For statistical analyses, unpaired student’s test, Mann–Whitney U test, Kruskal–Wallis test, and one-way ANOVA test were utilized. *P < 0.05; **P < 0.01; ***P < 0.001. [file 12967_2023_4760_MOESM1_ESM.tif]

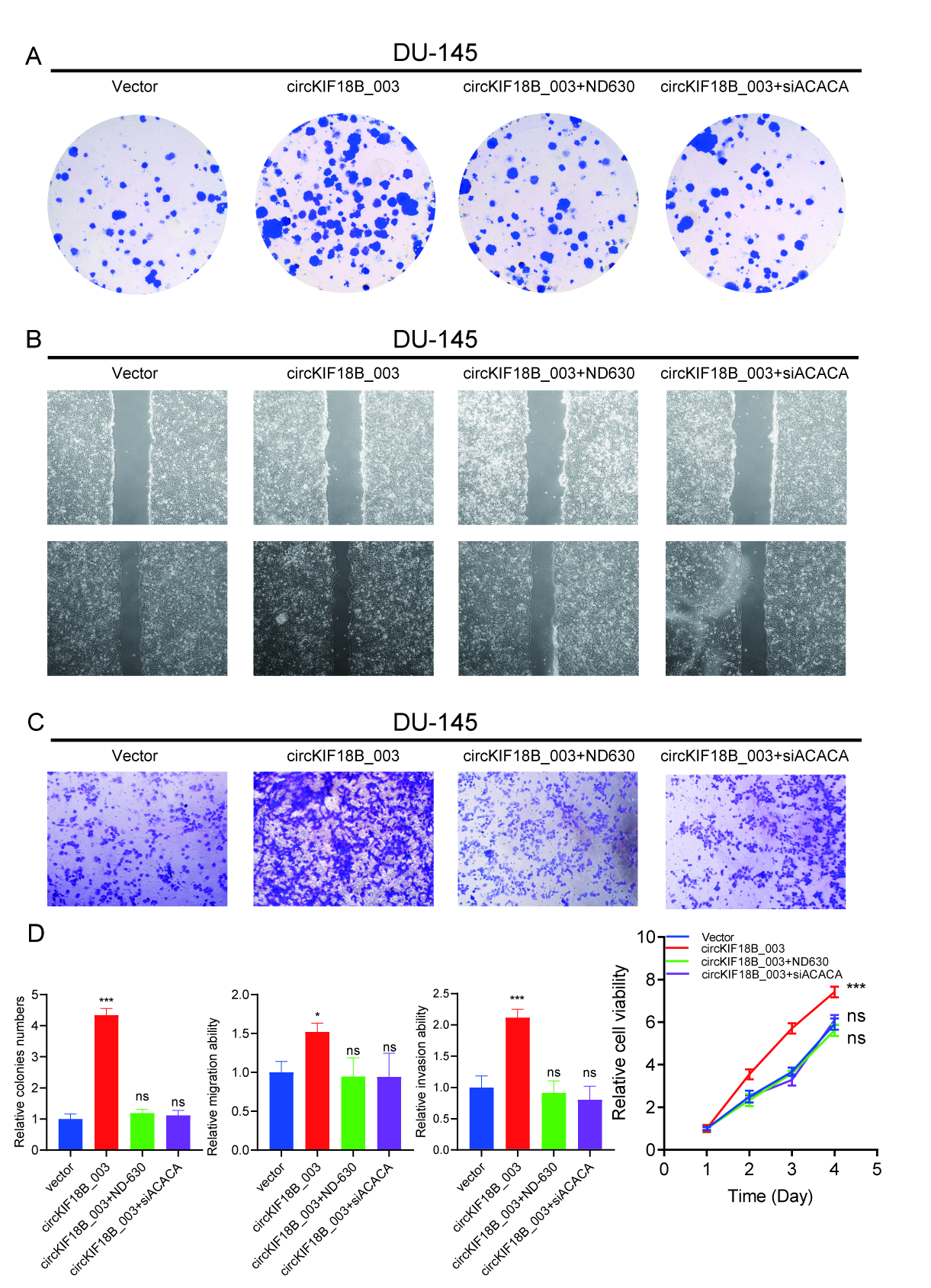

Supplement: Supplementary file 2 — Additional file 2: Figure S2. Influence of circKIF18B_003 on Cell Proliferation, Invasion, and Migration in DU145 Cell Line. A: Colony formation assay results showing that the circKIF18B_003 overexpression group showed the most significant increase in colony formation compared to the vector control group, thus indicating enhanced cell viability. The groups with circKIF18B_003 + ND630 and circKIF18B_003 + siACACA showed no significant changes in colony formation. B: Wound-healing migration assay results illustrating that overexpression of circKIF18B_003 resulted in the most noticeable increase in cell migration compared to the vector control group. The groups with circKIF18B_003 + ND630 and circKIF18B_003 + siACACA did not exhibit significant changes in cell migration. C: Transwell invasion assay results demonstrating that the circKIF18B_003 overexpression group had the highest increase in cell invasion compared to the vector control group. The groups treated with circKIF18B_003 + ND630 and circKIF18B_003 + siACACA did not display notable changes in cell invasion. D: Combined statistical graphs for colony formation assay, wound-healing migration assay, and transwell invasion assay results and CCK-8 assays, further confirming the most significant increase in cell viability in the circKIF18B_003 overexpression group compared to the vector control group. The groups with circKIF18B_003 + ND630 and circKIF18B_003 + siACACA did not show significant changes in cell viability. For statistical analyses, unpaired student’s test, Mann–Whitney U test, Kruskal–Wallis test, and one-way ANOVA test were utilized. *P < 0.05; **P < 0.01; ***P < 0.001. [file 12967_2023_4760_MOESM2_ESM.tif]

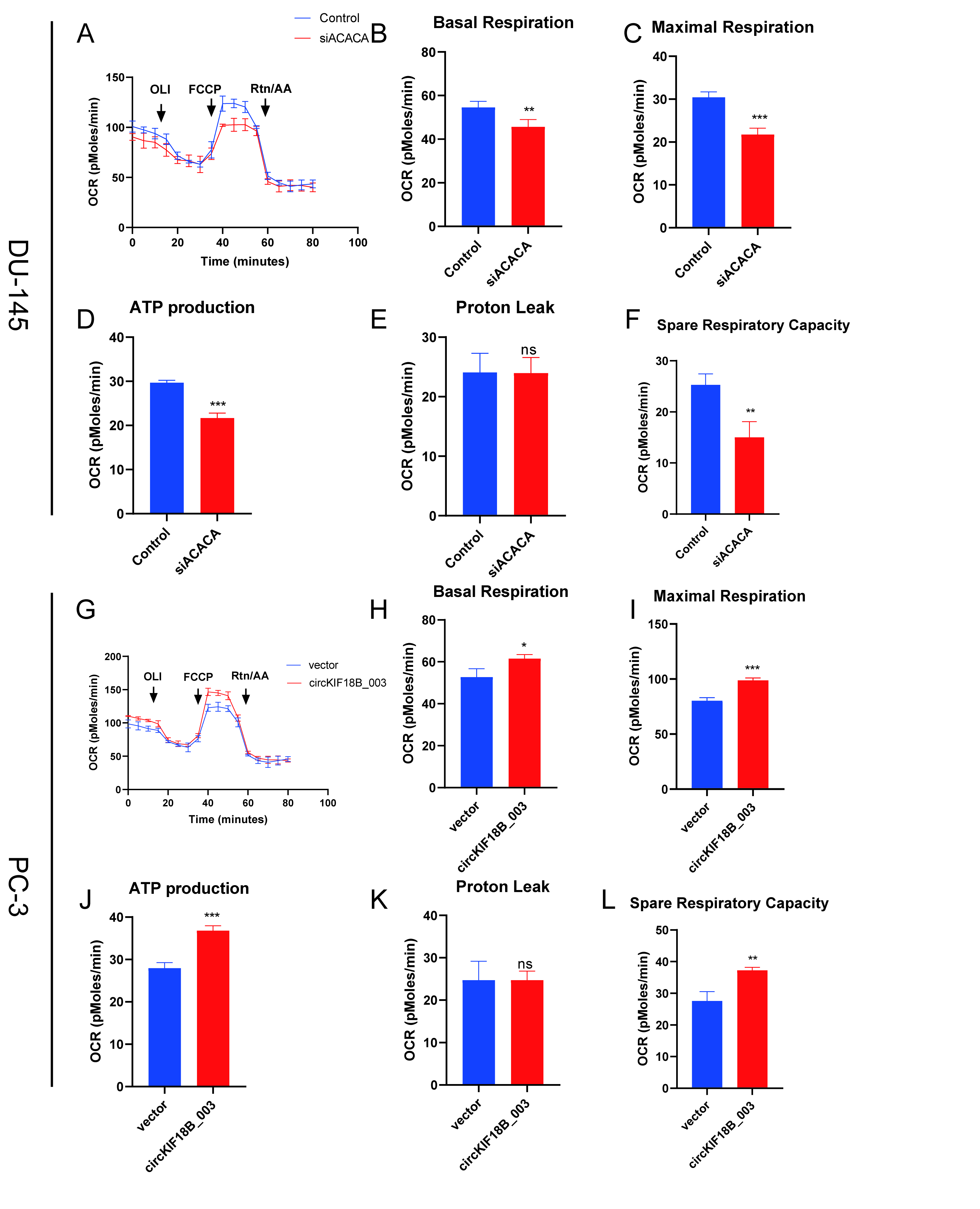

Supplement: Supplementary file 3 — Additional file 3: Figure S3. Metabolic changes in DU145 and PC-3 cell lines following ACACA downregulation and circKIF18B_003 overexpression. (A−F) Metabolic changes in the DU145 cell line due to ACACA downregulation. (A) Oxygen consumption rate (OCR) shows substantial alterations following ACACA downregulation. (B) Basal respiration and (C) maximal respiration are both significantly reduced in siACACA group. (D) ATP production is notably decreased in the siACACA group compared to the control. (E) Proton leak shows no significant difference between the two groups. (F) Spare respiratory capacity is reduced in the siACACA group, confirming the metabolic impact of ACACA downregulation. (G–L) Metabolic changes in the PC-3 cell line due to circKIF18B_003 overexpression. (G) OCR reveals a different pattern in the circKIF18B_003 group compared to the control. (H) Basal respiration, (I) maximal respiration, and (J) ATP production all show a significant increase in the circKIF18B_003 group. (K) Proton leak remains relatively consistent across groups. (L) Spare respiratory capacity is markedly elevated in the circKIF18B_003 group, indicating a higher metabolic potential. [file 12967_2023_4760_MOESM3_ESM.tif]
