# Supplementary material for: ND630 controls ACACA and lipid reprogramming in prostate cancer by regulating the expression of circKIF18B_003
Source: J Transl Med. 2023 Dec 4;21:877. doi: 10.1186/s12967-023-04760-w (PMC10694902; doi:10.1186/s12967-023-04760-w)
Supplement: Supplementary file 4 — Additional file 4: Table S1. Sequences of Primers used for RT-qPCR. [file 12967_2023_4760_MOESM4_ESM.docx]

**Table S1 Sequences of Primers used for RT-qPCR.**

| **circKIF18B_003** |  |
| --- | --- |
| F | 5’-AGGACACGTACAACACCCTC-3’ |
| R | 5’-ACCCGTACCACTACTTGCAG-3’ |
| **β-actin** |  |
| F | 5’-TGACGTGGACATCCGCAAAG-3’ |
| R | 5’-CTGGAAGGTGGACAGCGAGG-3’ |
| **ACACA** |  |
| F | 5’-AGACTGTGGTGGTTGGTAGA-3’ |
| R | 5’-CTGCTGGATTATCTTGGCTTCA-3’ |
| **miR-370-3p** |  |
| F | AATAATGCCTGCTGGGGTGGAA |
| R | AGTGCAGGGTCCGAGGTATT |
| RT | GTCGTATCCAGTGCAGGGTCCGAGGTATTCGCACTGGATACGACACCAGG |
