# Supplementary material for: ND630 controls ACACA and lipid reprogramming in prostate cancer by regulating the expression of circKIF18B_003
Source: J Transl Med. 2023 Dec 4;21:877. doi: 10.1186/s12967-023-04760-w (PMC10694902; doi:10.1186/s12967-023-04760-w)
Supplement: Supplementary file 5 — Additional file 5: Table S2. List of Primary Antibodies Used in the Study. [file 12967_2023_4760_MOESM5_ESM.docx]

**Table S2 List of Primary Antibodies Used in the Study.**

| Antibody | Applications | Company |
| --- | --- | --- |
| ACACA | WB, IF, IHC | Beyotime (AF6123) |
| p-AMPK | WB, IHC | Beyotime (AA393) |
| ACACA | WB, IP | CST (4190) |
| β-actin | WB, F, IF, IHC | CST (490) |
| AGO2 | WB, IHC, IP, CHIP, RIP | Abcam (ab32381) |
| IgG | WB, IHC, IP, CHIP, F | Abcam (ab172730) |

**Abbreviations:** WB, western blot; IHC, immunohistochemistry; IF, immunofluorescence; IP, immunoprecipitation; ELISA, enzyme-linked immunosorbent assay; F, flow cytometric analysis; RIP, RNA immunoprecipitation; CHIP, Chromatin Immunoprecipitation.
